# MSCF-LUNet: a lightweight three-stage pine wilt disease segmentation model with multi-scale context fusion mechanism

**DOI:** 10.3389/fpls.2025.1727626

**Published:** 2026-01-13

**Authors:** Dejing Zhou, Junxian Chen, Wenxi Cai, Jie Lin, Tiantian Meng, Yuanhang Li, Baihan Liu, Mengting Luo, Yubin Lan, Tianyi Liu, Jing Zhao

**Affiliations:** 1 College of Electronic Engineering, South China Agricultural University, Guangzhou, China; 2 Department of Computer and Information Science, University of Macau, Macau, Macao SAR, China; 3 The National Center for International Collaboration Research on Precision Agricultural Aviation Pesticides Spraying Technology, Guangzhou, China; 4 College of Forestry and Landscape Architecture, South China Agricultural University, Guangzhou, China

**Keywords:** complex forest stand environment, lightweight, multi-scale feature fusion, pine wilt disease, remote sensing image technology, segmentation

## Abstract

**Introduction:**

Pine wilt disease (PWD) is a highly destructive infectious disease that severely damages pine forests worldwide. Because symptoms emerge first in the tree crown, detection from unmanned aerial vehicles (UAVs) is efficient. However, most methods perform only binary classification and lack pixel-level staging, which leads to missed initial symptoms and confusion with other species.

**Methods:**

We propose MSCF-LUNet, a lightweight three-stage semantic segmentation model based on multi-scale context fusion. The model uses an improved multi-scale patch embedding guided by attention with relative position encoding (AWRP) to adapt the sampling field of view and to fuse local details with global context. Under contextual attention, the network learns fine-grained features and location cues.

**Results:**

In complex environments, MSCF-LUNet achieves 89.56% precision, 92.13% recall, 88.92% intersection over union (IoU), and 96.54% pixel accuracy (PA), balancing performance and computational cost.

**Discussion:**

The model effectively segments PWD-infected regions and determines disease stages from remote-sensing imagery.

## 1 Introduction

Pine species play a key role in afforestation on degraded land. They protect soil and support water conservation. They also have high carbon sequestration potential, which is important for ecological restoration of degraded land worldwide (Tudor et al., 2025). PWD is a highly destructive forest disease caused by the pinewood nematode (PWN, Bursaphelenchus xylophilus). In 1972, Mamiya and Kiyohara first confirmed its pathogenic mechanism (Mamiya and Kiyohara, 1972). Since then, PWD has spread rapidly in Asia and Europe and has become one of the most serious forest diseases in these regions. Many countries now list PWD as a quarantine pest, and China is among the most severely affected countries (Li et al., 2011a). In 2024, the National Forestry and Grassland Administration (NFGA) of China issued the Technical Plan for the Prevention and Control of Pine Wilt Disease (NFGA, 2024). This plan refers to PWD as the “cancer of pine trees” because it has a mortality rate of nearly 100%. PWD can kill infected pine trees within about 40 days and can destroy entire pine stands within about 3 years. The development of PWD can be divided into three stages: the initial stage, the intermediate stage, and the advanced stage (Jung et al., 2024). Stage-specific monitoring can increase the efficiency of prevention and control by more than 300% (NFGA, 2024). The initial stage is especially important, because control at this stage can block further spread. During the initial stage, the targeted removal of infected trees can stop the disease and avoid the high felling costs that occur when whole stands die in the intermediate and advanced stages. Previous studies mainly focused on pathogen detection and laboratory-based assays for PWD. For example, Zhao et al. (2005) designed specific primers for the rDNA-ITS1 region and used PCR to detect single individuals of Bursaphelenchus xylophilus and Bursaphelenchus mucronatus. However, this method depends on specialized laboratory equipment and DNA extraction, so it does not meet the needs of real-time field monitoring.

To overcome the limitations of traditional methods and enable large-scale field monitoring, many researchers have used remote sensing imagery. Pan et al. (2024) addressed the unclear initial symptoms of PWD by regularly collecting hyperspectral data with ground-based non-imaging spectrometers and UAV-borne imaging spectrometers. They established a technical framework for initial remote sensing monitoring of PWD. Deng et al. (2020) used UAVs equipped with global positioning system (GPS) modules and achieved more than 90% accuracy for binary detection of dead trees. Their method also provided the geographic coordinates of each dead tree. However, such binary classification models only distinguish “diseased” and “healthy” pine trees in pure pine stands and cannot identify the initial, intermediate, and advanced stages of PWD. This limits their practical use. Yu et al. (2021) combined UAV hyperspectral imagery (HI) and light detection and ranging (LiDAR) data and achieved three-stage classification at the individual-tree scale using a Random Forest (RF) algorithm (overall accuracy: 73.96%; Kappa coefficient: 0.66). This approach improved the recognition of multiple disease stages, but the spectral signatures of initial diseased trees and healthy trees still strongly overlap. As a result, the misclassification rate for the initial stage remains high, which restricts its application in initial prevention and control. Iordache et al. (2020) also used an RF classifier with hyperspectral and multispectral data and achieved more than 91% overall accuracy. However, from the intermediate stage onward, diseased trees show subtle changes in canopy spatial structure, such as branch and leaf distribution and canopy density. In their implementation, the RF model mainly relied on spectral features and could not effectively capture this structural information. This led to lower recognition accuracy for the intermediate and advanced stages and did not fully meet the requirements of multi-stage detection. In addition, spectral analysis methods require large, well-labeled training datasets, which increases data processing and annotation costs. Currently, Two challenges limit binary classifiers (Jung et al., 2024): First, initial and intermediate-stage browning is often confused with normal defoliation. Second, advanced-stage withering must be distinguished from similar patterns in other species. How to realize more efficient semantic segmentation of the characteristics of PWD’s initial, intermediate stage, and advanced stage in the remote sensing vision is currently a research focus in the field of forestry artificial intelligence.

In recent years, deep learning has provided a new paradigm for disease identification in remote sensing. Several studies have shown that lightweight models can detect diseases and pests from high-resolution imagery. For example, Du et al. (2024) replaced the backbone network of YOLOv5 with ShuffleNetV2 and achieved accurate binary detection of PWD under lightweight settings. However, this method depends on high-resolution datasets and pure pine forest environments, so it is sensitive to interference from other environmental features. Yuan et al. (2025) proposed YOLOv8-RD, a lightweight model for PWD detection in complex and interfering environments, and Wang et al. (2024) proposed YOLO-PWD for simpler environments. YOLOv8-RD improves robustness through feature concatenation, while YOLO-PWD reduces the computational burden on edge devices. Both models mainly identify pine trees with obvious discoloration in the intermediate and advanced stages and perform poorly for trees in the initial stage, where discoloration is weak and feature interaction is limited. Xu et al. (2025) proposed the FIDC-YOLO model, which enhances feature interaction and feature capture, improves detection performance, and supports faster deployment. However, this method still performs only binary classification of “diseased trees” and “healthy trees” and does not provide fine-grained segmentation of pine trees at multiple disease stages. This limits its practical value in forestry remote sensing prevention and control. Overall, excessive pursuit of lightweight design can reduce detection reliability in complex real-world environments and hinders accurate localization and perception of PWD at different stages. Segmentation algorithms that balance lightweight design and detection performance are therefore crucial. Zhang, Yao, and Dong’s teams built a reliable UAV-based framework for PWD detection and verified it with field data. (Zhang et al., 2025), (Yao et al., 2024), (Dong et al., 2024), They upgraded the YOLO model step by step. The results show that task-specific loss functions and improved feature fusion and extraction boost detection accuracy. The work also highlights the promise of collaborative hybrid loss functions for remote-sensing detection.

In fine-grained segmentation, UNet has shown strong local feature extraction ability in many image segmentation tasks because of its encoder–decoder structure (Ronneberger et al., 2015). Shen et al. (2024) fused small VGG convolution kernels with UNet, reducing parameters and enlarging the receptive field. However, their method still has limited accuracy for initial-stage diseased pine trees with slight canopy discoloration and does not meet the needs of disease initial warning. This suggests that single-branch semantic structures lack effective multi-scale feature collaboration for pine wilt disease. Vision Transformer (ViT) is efficient for global image feature extraction and uses global attention to capture canopy-scale wilting patterns (Dosovitskiy et al., 2021). The Clusterformer model proposed by Liu et al. (2024) breaks the local receptive field limitation of traditional convolutional networks through the Cluster Token Mixer and Spatial-Channel Feed-Forward Network (SC-FFN), and greatly improves disease segmentation accuracy under complex backgrounds. However, its Transformer-based decoder has high computational complexity, which restricts deployment in resource-constrained and edge scenarios. In addition, Transformer architectures may show limited sensitivity to subtle initial- and late-stage features of a single disease such as pine wilt disease.

When ViT and UNet are fused in a simple and efficient way, layer-wise fusion of global semantics and scene features can significantly improve segmentation accuracy in complex settings (Zhou et al., 2024). Mainstream models such as Swin-Unet (Cao et al., 2021) and TransUNet (Chen et al., 2021) integrate ViT or its variants into UNet-like structures. For example, TransUNet introduces

global self-attention into convolutional networks to enhance both local and global semantic modeling in segmentation. The proposal of PKINet (Cai et al., 2024) further shows that long-range context dependencies can strengthen central feature representations of valid samples and improve detection performance in remote sensing images. These results indicate that combining global semantic information with contextual perception is effective for handling multi-scale targets and confusing small objects. Motivated by these findings, this study proposes a lightweight segmentation model guided by a multi-scale context fusion mechanism to improve rapid perception of multiple disease stages from a remote sensing perspective. While enhancing single-branch feature transmission, we design a lightweight dual-branch dynamic feature interaction mechanism for local details and global context information. This dual-branch structure refines feature perception and fusion and improves sensitivity to disease features at different stages. We fuse an improved multi-scale sampling mechanism with UNet in a layer-wise manner under lightweight context attention, and name the overall mechanism Multi-scale Context Fusion (MSCF). When combined with a lightweight UNet, the model is called MSCF-LUNet. MSCF-LUNet aims to achieve fine-grained disease region localization and stage-specific perception for multi-stage disease segmentation in complex forest remote sensing scenes while maintaining high robustness. The advantages and innovations of semantic segmentation from the remote sensing perspective are as follows:

### 1.1 Complementary advantages

Remote-sensing scenes mix small targets with broad context. To meet speed and accuracy needs, we keep the strengths of UNet and Transformers. UNet serves as the backbone and focuses on stage-specific, fine details. In parallel, a multi-scale extractor captures global scene semantics. The MSCF module links shallow, mid, and deep UNet features with the global branch, sharpening blurred cues and aligning local detail with context. The model stays sensitive to targets across scales without a heavy compute load.

### 1.2 Branch integration

A dual-branch parallel approach is introduced to achieve dimensional alignment and intersection fusion of features while reducing detail loss. With reasonable compression, Depthwise Separable Convolutions (DSC) replace standard convolutions. Dilated convolutions enlarge the receptive field while preserving lesion edges, lowering cost yet covering tree and background cues. Learned multi-scale patch embedding and cross-scale relative-position encoding operate under Light Context Attention (LCA). This setup uses inter-tree environment signals and strengthens long-range links between disease patterns and background. False positives from non-target trees and similar look-alikes drop in both dense and sparse stands. Sampling scale adapts to image detail, avoiding redundant noise and improving perception of target appearance and spatial layout.

### 1.3 Feedback optimization

A hybrid loss function including Focal Loss (Lin et al., 2020), Tversky Loss (Wang et al., 2017), and Boundary Loss (Kervadec et al., 2021) is introduced: the focus of the loss function is dynamically adjusted according to the inter-tree density. The loss focus is adjusted by inter-tree density. Coupled with the fusion design, segmentation of the three disease-feature types improves, and feature extraction remains reliable across scales in complex scenes.

In summary, MSCF-LUNet adapts well to varied environments. In forestry remote sensing, it can locate affected areas and separate disease stages quickly and accurately, enabling timely, targeted intervention and reducing losses.

## 2 Dataset description and processing setup

**Figure 1:**
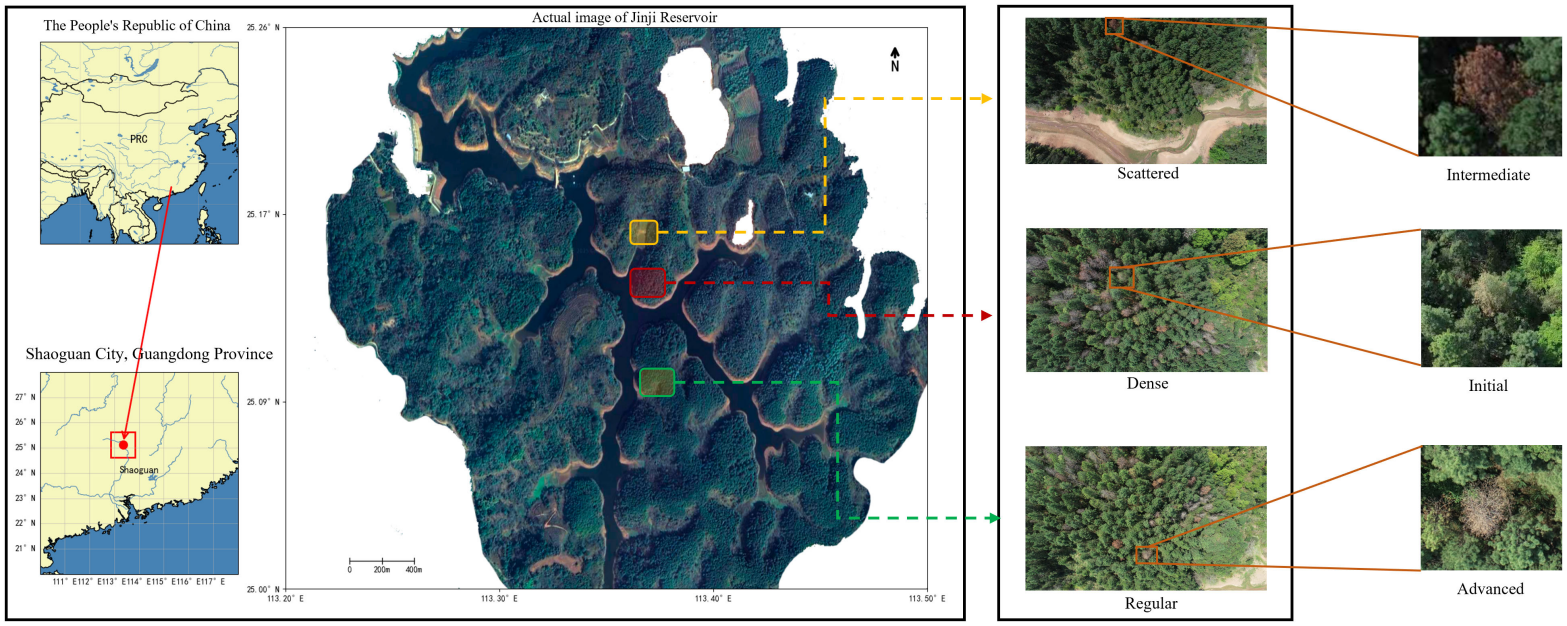
Decline characteristics of pinus elliottii infected by PWD and overview of the study area. The diseased trees were split by three stages and UAV sampled them in diverse scenarios, with a sampling height of 80 meters above the takeoff location and a processing resolution of 600x800.

This study focuses on identifying and monitoring PWD in pine trees at three stages: initial, intermediate, and advanced. The dataset was collected in the Jinji Reservoir area of Lechang, Shaoguan, Guangdong, China (113.45°E, 25.07°N). The site is a typical mountain reservoir environment in South China. Pinus elliottii (slash pine) is the dominant vegetation and the main host of pine wilt disease in this region. In this area, the disease shows clear stage specific decline patterns, as shown in **Figure 1**. In the initial stage, a small number of yellow spots appear on needles or some needles turn pale yellow. In the intermediate stage, needles are completely yellow with no green, with partial shedding of branches and needles. In the advanced stage, all needles fall and branches become gray brown. These differences guided data collection and the division of disease stages.

Due to the spectral limitations of RGB imagery, the accurate identification of initial-stage PWD was particularly difficult. To improve the reliability of ground-truth labels, we designed a structured workflow. First, we conducted preliminary UAV flights to capture aerial images and identify suspected infected trees. From these candidates, we selected representative samples that showed visual features easily confused with initial PWD symptoms. Field surveys were then carried out to inspect these trees and confirm the presence of pinewood nematode infestation. This step helped establish a set of validated diagnostic characteristics visible in RGB images.

Meanwhile, there is no large-scale industrial pollution or human disturbance around the reservoir, so the growth status of pine trees affected by PWD can truly reflect the evolutionary law of PWD in natural environments. To evaluate the performance of the MSCF-LUNet in the multi-stage identification of PWD in complex and pristine ecological environments, this study adopted a color RGB image dataset captured *via* high-altitude overhead photography using a DJI Phantom 4 RTK UAV. The UAV is equipped with a 1-inch 20-megapixel CMOS sensor and an 84° wide-angle camera. RGB images captured at noon time from April to July 2025 at an altitude of 80 meters were selected, which basically cover the slash pine green areas in the reservoir area. During labeling, we used Labelme to draw polygons around diseased trees and assign PWD stage labels based on field-verified rules. Images found to be blurry during annotation were removed. To keep labels consistent, one annotator did the first pass. Multiple reviewers then checked the labels, and a group of forestry experts resolved disagreements. The final dataset contains 21,300 diseased-tree samples, split 70/10/20 into training/validation/test. Counts and class distribution are shown in **Table 1**.

**Table 1:** Dataset segmentation (split 70/10/20 into training/validation/test).

Dataset	Initial	Intermediate	Advanced
Training set	4532	4799	5577
Validation set	647	685	796
Test set	1297	1373	1594
Total	6476	6857	7967

**Table 2:** Results of MSCF-LUNet ablation experiment.

Model	mP (%)	mR (%)	mIoU (%)	mPA (%)	Parameters (M)	FLOPs (G)	Weight (MB)
UNet	84.56	85.37	85.14	94.82	31.25	399.13	119.26
LUNet	80.12	80.43	79.27	87.64	7.71	24.77	19.61
LUNet+DSC	79.28	79.97	78.42	86.04	4.52	17.81	15.56
LUNet+LCA	81.71	82.56	80.32	92.89	8.31	24.86	20.63
LUNet+DSC+LCA	80.28	82.74	81.84	91.56	7.10	20.90	16.58
LUNet+MSF	84.85	85.67	86.42	95.07	24.29	69.04	92.19
LUNet+DSC+MSF	85.45	85.72	85.62	93.15	21.86	61.08	86.14
LUNet+LCA+MSF	88.95	91.62	88.53	**96.72**	25.14	71.13	93.21
MSCF-LUNet	**89.56**	**92.13**	**88.92**	96.54	23.28	64.17	89.16

optimal value

Before training, each image was resized to 600 × 800 pixels, and the corresponding mask was resampled using nearest-neighbor interpolation to preserve label integrity. During training, we applied MixUp with ɑ; = 0.4 and CutMix with ɑ; = 0.4 on mini-batches. MixUp forms a convex combination of two images and their masks using a Beta(ɑ;,ɑ;) weight, and the supervised signal is the same convex combination of the two masks. CutMix replaces a randomly sampled rectangular region of one image with the corresponding region from another image; the target mask is updated by inserting the same region from the donor mask, and the per-pixel supervision follows this composite. At inference, the model runs in evaluation mode on single-scale 600 × 800 inputs. Pixel labels are obtained by taking the argmax over class scores, which yields a deterministic output for a given input.

## 3 Method

**Figure 2:**
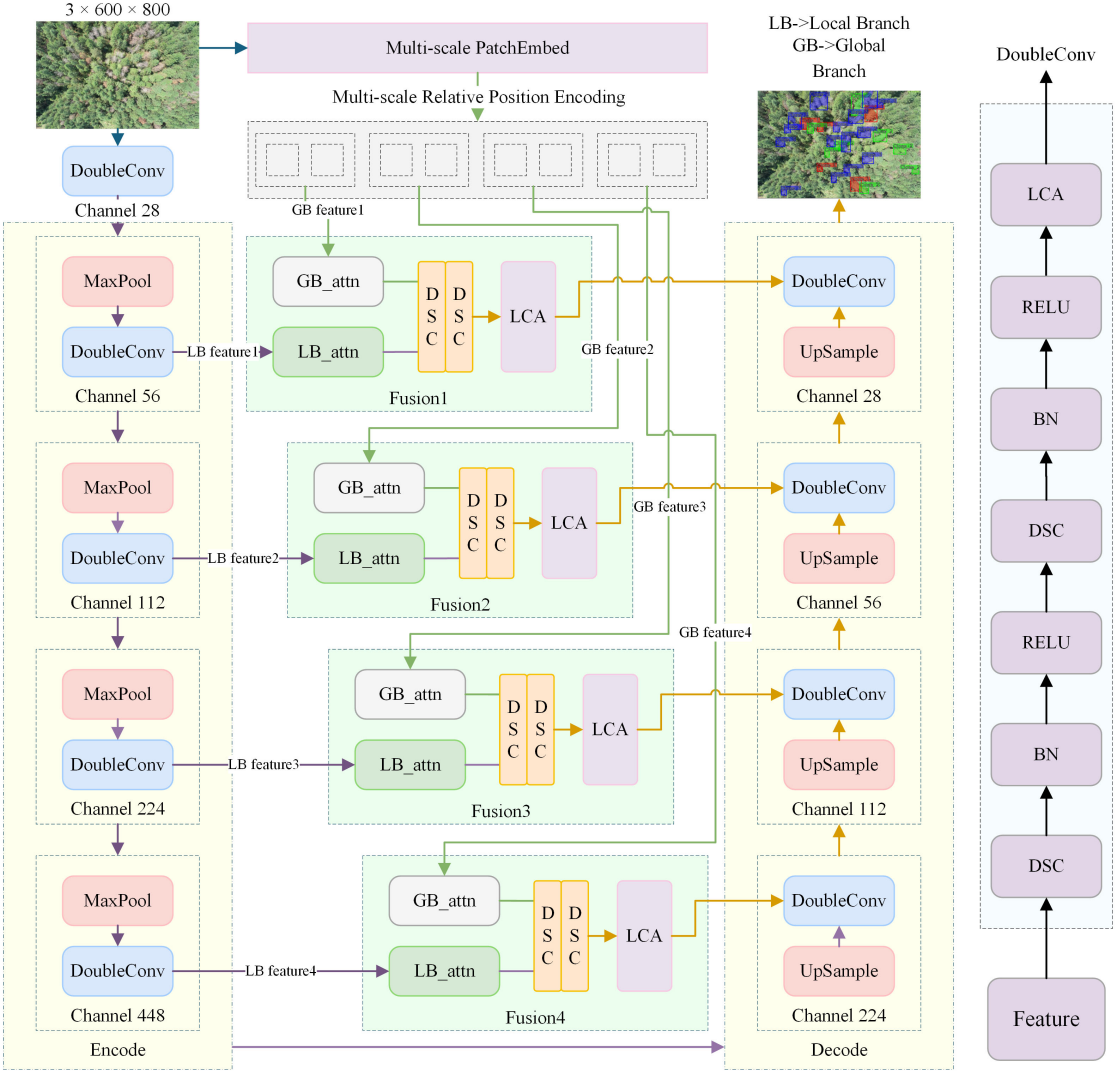
MSCF-LUNet lightweighting is primarily achieved through model pruning compression and optimization of convolutional modules. When integrated with the multi-scale context fusion mechanism, it forms the framework for model feature extraction and segmentation.

Remote sensing of PWD in pine forests faces two core problems: subtle early symptoms and heavy scene interference. Confusing features, motion blur, jitter, and crown occlusion all make detection harder. We address this with MSCF-LUNet. The model pairs UNet for fine local cues with a ViT patch-embedding path for global context. Their features are fused with context attention and multi-scale enhancement, then sent to the decoder. We add ArcFace (Deng et al., 2022) for scene-level classification and a hybrid loss for segmentation. Together, these raise accuracy at both image and pixel levels. The system delivers end-to-end, stage-level pixel-wise segmentation and supports large-area status assessment. It improves confidence in early warnings and helps locate and manage infected stands. The overall model architecture is shown in **Figure 2**.

### 3.1 Overall architecture of MSCF-LUNet

MSCF-LUNet uses an encoder–decoder with cross-scale feature fusion, tailored to 600×800 UAV RGB images for PWD. The model follows a collaborative multi-task setup with hierarchical feature interaction. A modified UNet acts as the local segmentation branch and extracts stage-specific cues and localizes lesions. In parallel, a ViT-style patch-embedding path, upgraded for multi-scale processing, serves as the global classification branch to model scene context and rate overall disease severity. The two branches interact through an attention-guided feature fusion (AGFF) module. High-level semantics from the ViT path are merged with UNet encoder features *via* spatial and channel attention. The fused features are injected into multiple UNet decoder stages. During joint training, global semantics are fed back to the encoder for self-correction and enhancement. This design improves separation of blurred or low-contrast cues, increases robustness to environmental interference, and enables accurate lesion localization, fine segmentation, and correct scene-level classification.

### 3.2 Improved local feature enhancement backbone network

The encoder part of the original UNet is designed to capture local discriminative features of different PWD stages under large-scale complex backgrounds, thereby improving the ability to distinguish details of features at various scales. UNet consists of an initial convolutional block and 4 downsampling modules. In this study, lightweight and performance optimization improvements are made to the UNet network: first, model pruning and channel compression methods (Zhou et al., 2018) are used to reduce the network depth to avoid the gradient vanishing; second, edge-enhanced DSC is added to the backbone network, forming a double-layer lightweight convolution to replace the original backbone network’s convolutional blocks; finally, a lightweight context attention mechanism is introduced, resulting in a backbone network suitable for tasks with complex backgrounds.

#### 3.2.1 Edge-enhanced DSC

Traditional standard convolution incurs high computational cost when processing 600×800 high-resolution remote sensing images. To strike a balance between efficiency and feature extraction capability, this study constructs a new edge enhancement branch by fusing DSC with reduced original convolutions. This design effectively reduces the number of model parameters while preserving as much edge detail required for PWD lesion localization as possible.

Image features are first processed through depthwise convolution, where a 3×3 convolution is independently applied to each input channel to capture spatial correlations within a single channel. For scenarios requiring a larger receptive field, a dilated convolution with dilation=2 is adopted to expand the field of view without increasing parameters, thereby focusing on more feature regions. Its mathematical expression is:

(1) $$\begin{array}{c} X_{depthwise}=Conv_{k=3\;},\;g=C_{in},\;\;d=dil(X_{in}) \end{array}$$

In Equation 1, ${\text{X}}_{depthwise}$ denotes the output of the depthwise convolution, ${\text{C}}_{in}$ is the number of input channels, dil represents the dilation rate, ${\text{X}}_{in}$ is the input feature, and $\text{g}={\text{C}}_{in}$ indicates grouped convolution. The second stage is pointwise convolution, which fuses cross-channel information *via* 1×1 convolution and adjusts the number of channels to the target output dimension. The expression is:

(2) $$\begin{array}{c} X_{pointwise}=Conv_{k=1\;}(X_{depthwise}) \end{array}$$

In Equation 2, ${\text{X}}_{pointwise}$ is the output of the pointwise convolution. Finally, the output of the pointwise convolution is processed through a 3×3 depthwise convolution to strengthen edge features (e.g., boundaries between healthy and diseased leaves). The enhanced edges are superimposed back onto the output of the pointwise convolution with a weight of 0.2 to avoid excessively overshadowing the main features. The specific expression is:

(3) $$\begin{array}{c} \;X_{edge}=Conv_{k=1\;},g=C_{out}(X_{pointwise}),X_{out}=X_{depthwise}+0.2\times X_{edge} \end{array}$$

In Equation 3, ${\text{ C}}_{out}\;$ is the number of output channels, ${\text{X}}_{edge}$ represents edge features, and ${\text{ X}}_{out}$ denotes the total output features.

### 3.3 Light context attention

Context attention is widely used in multi-scale perception tasks (Ouyang et al., 2023), especially in detection and segmentation under complex and confusing backgrounds. In initial-stage PWD, disease symptoms are often subtle. Compared with intermediate and advanced stages, small areas of necrosis or leaf yellowing can be mistaken for normal forest changes, leading to missed detections or misclassification of healthy trees as diseased ones. In this study, we proposed a LCA module, which adapts to different receptive fields and enhances spatial positioning and structure awareness. The module improves perception of disease-related features while suppressing background noise and strengthening long-range relationships among similar disease patterns. This allows the model to distinguish small and easily confused targets more effectively, providing key technical support for accurate extraction and segmentation of multi-scale PWD features.

To avoid the model’s overfitting to local information or information redundancy, context attention is mainly formed by fusing lightweight spatial and channel attention. At this point, channel attention focuses on enhancing channels containing PWD discriminative information, with primary emphasis on color features that are sensitive to disease characteristics. Through Global Average Pooling (GAP) (Lin et al., 2014) and Global Max Pooling (GMP) (Zhou et al., 2016), the spatial dimension of the input feature map is compressed to 1×1, capturing the mean and extreme value statistical information of channels respectively. Their expressions are as follows:

(4) $$\begin{array}{c} \;x_{avg}=GAP\left(x_{in}\right)=\frac{1}{H\cdot W}\sum_{i=1}^{H}\sum_{j=1}^{W}x_{in}\left(i,j,:\right) \end{array}$$

(5) $$\begin{array}{c} x_{max}=GMP\left(x_{in}\right)=\underset{i=1.H,j=1.W}{max}x_{in}\left(i,j,:\right) \end{array}$$

In Equations 4, 5, *H* and *W* represent the height and width of the input feature map, respectively. Subsequently, the pooled features are processed through a two-layer bottleneck structure of “1×1 convolution → ReLU → 1×1 convolution” to generate channel attention weights, thereby reducing computational complexity. The expressions are as follows in Equations 6, 7:

(6) wavg=DSC(ReLU(DSC(xavg)))

(7) wmax=DSC(ReLU(DSC(xmax)))

Finally, the final channel attention weights are obtained *via* the sigmoid activation function and element-wise addition, and these weights act on the input feature map. The specific expressions are:

(8) $$\begin{array}{c} w_{channel}=\sigma \left(cat(w_{avg},w_{max})\right)x_{channel}=x_{in}⊙w_{channel} \end{array}$$

In Equation 8, *σ* denotes the sigmoid function, ⊙ represents element-wise multiplication, and cat refers to residual connection. Meanwhile, max pooling and average pooling operations are performed along the channel dimension to compress multi-channel information into single-channel features, reducing cross-channel differences in intensity distribution (e.g., details of diseased areas and background environments). After concatenation, a 2-channel feature map is generated to capture cross-channel intensity changes, with the expression as follows:

(9) $$\begin{array}{c} w_{Cross}=DSC\left(DSC\left(cat({max}_{c}\left(x_{channel}\right),mean_{c}\left(x_{channel}\right)\right)\right) \end{array}$$

(10) $$\begin{array}{c} \partial =\sigma \left(Conv_{1\times 1}\left(mean_{c}\left(x_{channel}\right)\right)\right) \end{array}$$

In Equations 9, 10, ${max}_{\text{c}}$ and ${mean}_{\text{c}}$ represent max pooling and average pooling along the channel dimension, respectively, and $w_{Cross}$ denotes the cross-channel aggregation weight. Subsequently, the two types of weights are fused, and an adaptive adjustment factor ∂ is introduced to adapt to the perception and attention capabilities under different disease scenario densities, as well as conditions such as feature overlap and occlusion. The expression is as follows:

(11) $$\begin{array}{c} \;x_{attn}=x_{channel}⊙w_{Cross}\times \partial \end{array}$$

**Figure 3:**
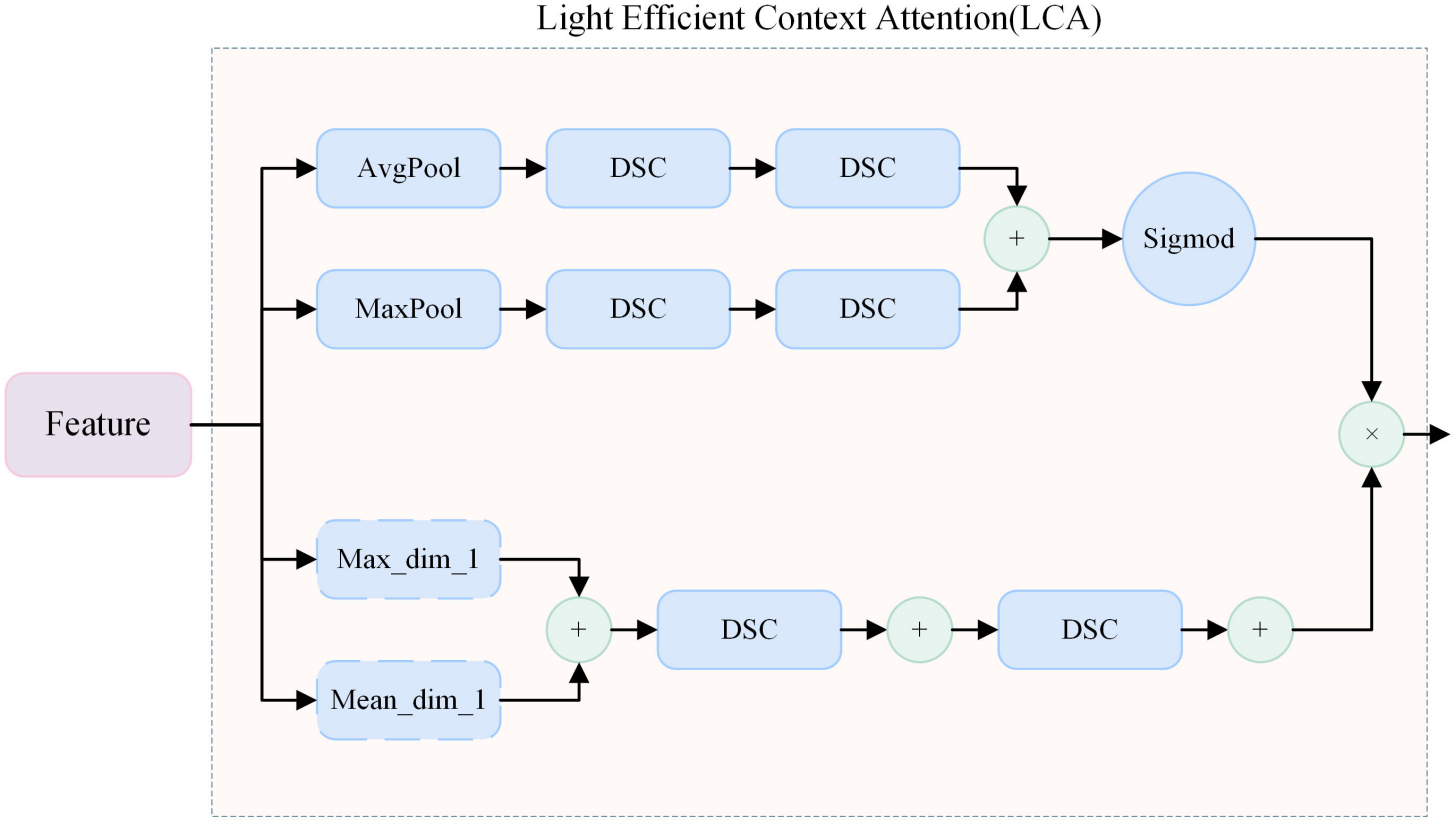
Detailed structure of the proposed Light Context Attention module.

In Equation 11, $x_{attn}$ is the adaptive output of mixed attention. The context attention mechanism ultimately forms part of the dual convolution block together with DSC. Meanwhile, information transmission is conducted during downsampling and feature fusion, strengthening the long-range contextual correlation among various features in the local space under receptive fields of different scales. When locking onto diseased areas, it focuses on the surrounding environment of the target area and adjusts the focus weight based on global semantic information. On the premise of not introducing excessive noise, it effectively improves the feature information fidelity of diseased trees at various stages in complex backgrounds. The details of the module are shown in **Figure 3**.

### 3.4 Multi-scale feature extraction branch

To connect the advantage of the improved UNet encoder in enhancing local features and make up for the model’s limitations in global semantic information extraction, this study extracts the encoder component from the ViT to process the global semantic information of image features. Meanwhile, to maintain an as lightweight model architecture as possible, a learnable Multi-scale Patch Embedding mechanism is designed to introduce multi-scale space into the feature sequence representation and control the sequence length. This mechanism combines a positional encoding module with 2D relative positional attention to provide “distance-direction” geometric constraints for features, enabling higher prior similarity among adjacent regions of the same tree crown. Ultimately, this improves the segmentation perception ability of PWD and reduces interference from similar features and structures of other tree species.

#### 3.4.1 Multi-scale patch embedding

**Figure 4:**
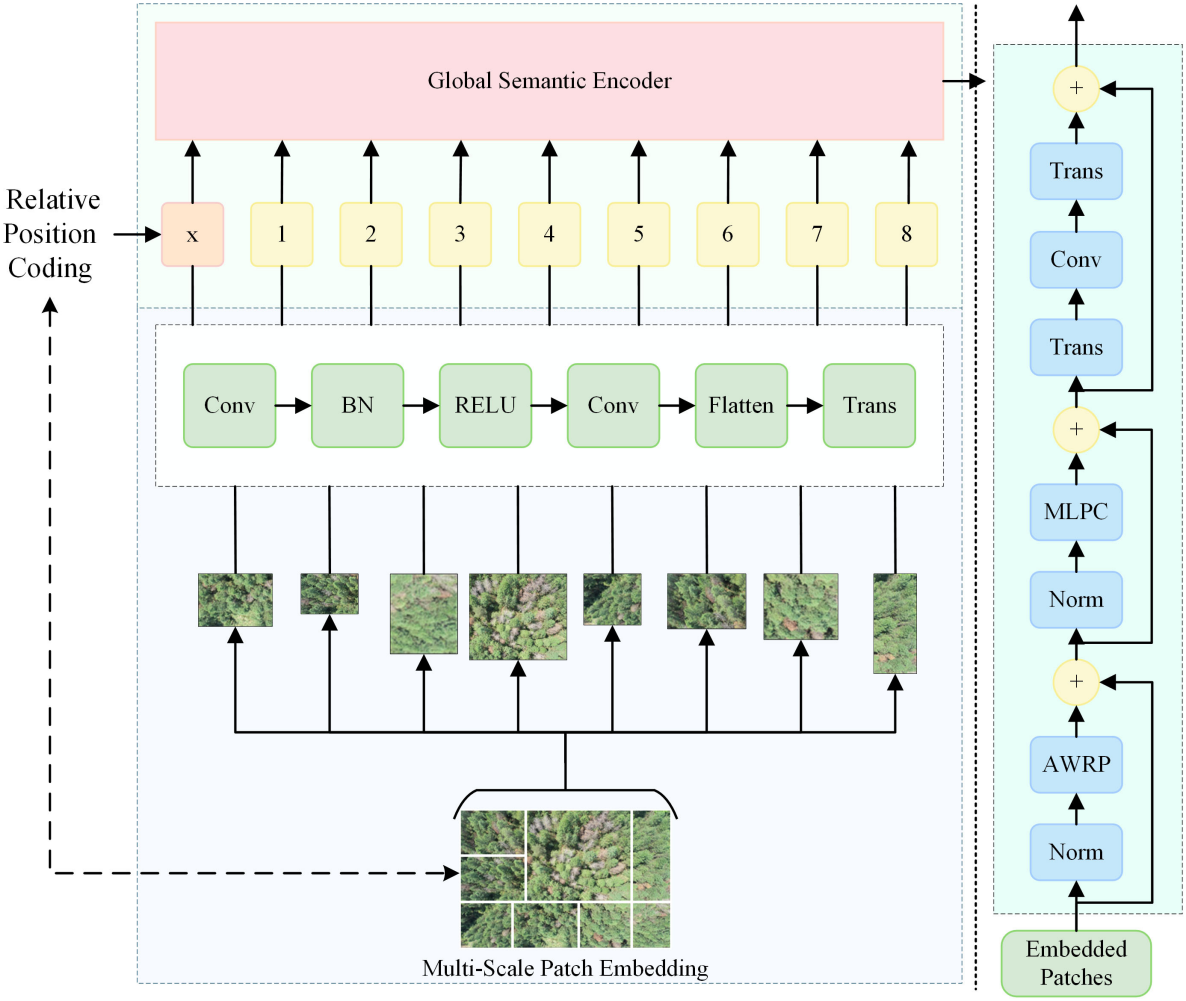
Detailed structure of the proposed Multi-scale Patch Embedding module.

Although the traditional ViT adopts fixed-size patches for linear projection, which is highly effective in expanding the spatial perception performance of semantic information, it still lacks the continuity of detailed parts. Moreover, it tends to result in excessively long sequences, thereby ignoring the existence and features of aliased semantic information, which further leads to local optimality and performance overhead. This study proposes a learnable Multi-scale Patch Embedding. On the basis of two-stage convolutional embedding, a learnable adaptive multi-scale patch embedding is introduced: through multi-scale lightweight convolution branches and a learnable router, each patch adaptively selects the optimal scale, and the step size is adjusted to control the sequence length, thus processing feature details in the most efficient manner. Its structure is shown in **Figure 4**.

Two-stage convolutional embedding processing (Wu et al., 2021) is a mainstream sampling method. In this study, the input image is first sampled and compressed on features using multi-scale convolution kernels with different step sizes. Then, a 1×1 convolution kernel is used to uniformly map the image to the embedding dimension, completing the patch-based mapping and outputting feature details. The processing flow is as follows:

(12) $$\begin{array}{c} U=ReLU\left(BN\left(Conv_{k_{i}}^{s_{i},p_{i}}\left(I\right)\right)\right)\in R^{D\times H\times W} \end{array}$$

(13) $$\begin{array}{c} E_{i}=Conv_{1x1}\left(U\right)\in R^{D\times H_{i}\times W_{i}} \end{array}$$

In Equations 12, 13, i denotes the number of scale branches, *D* is the ViT embedding dimension, the superscripts ${\text{p}}_{\text{i}}$ and ${\text{s}}_{\text{i}}$ represent padding and step size, respectively, the subscript denotes the size of the convolution kernel, and *H* and *W* are the height and width of the input image. According to the imported image and feature details, the patches adaptively adjust their sizes to match the changes in feature distribution, learn more abundant local feature details ${\text{E}}_{\text{i}}$, and apply neighbor low-pass after alignment to suppress aliasing and noise. At this point, the introduced learnable router generates scale weights ${\text{π}}_{\text{i}}\left(h,w\right)$ for each position after processing with the temperature coefficient τ and softmax. The calculation formula is as follows:

(14) $$\begin{array}{c} \pi_{i}\left(h,w\right)=soft{max}_{i}\left(g_{i}\left(x\right)\in R^{i\times h\times w}/\tau \right)\;h=H/s_{i}\;\;w=W/s_{i} \end{array}$$

In Equation 14, τ is adjusted according to the scene density, and ${\text{g}}_{\text{i}}\left(x\right)$ is a feature router that generates scale weights and U is for the original patch positions. Finally, it is fused with the feature details $E_{i}\left(h,w\right)$:

(15) $$\begin{array}{c} \;Y\left(h,w\right)=\sum_{i=1}^{8}\pi_{i}\left(h,w\right)-E_{i}\left(h,w\right) \end{array}\;$$

In Equation 15, Y(h,w) represents the information of multi-scale features after adaptive learning. Its value is averaged, and the difference is calculated by comparing it with the feature values of patches at various scales. This difference is used as a weight to feedback and dynamically adjust the convolution kernel specifications and the size of each patch. As training progresses, the patch size is gradually adjusted to include the real disease features in each patch to the maximum extent for subsequent processing. Finally, after flattening in the vector direction, it is concatenated with the class token and superimposed with the sine value of positional encoding to obtain the sequence representation:

(16) $$\begin{array}{c} {\bar{Z}}_{p}=\left[e_{cls};vec\left(Y\right)\right]\;x=\pi_{p}\left(h,w\right) \end{array}$$

In Equation 16, ${\bar{Z}}_{p}$ is the sequence representation, p is the number of each patch, $e_{cls}$ is the class token, $\left[e_{cls};vec\left(Y\right)\right]$ denotes concatenation along the sequence dimension, and finally the token vector of a certain class is concatenated to the head of the sequence of all patch vectors. Compared with single linear patchification, the two-stage embedding method effectively reduces the interference of single sequence information on the model’s overall feature extraction and avoids image information distortion. Meanwhile, this method can flexibly obtain more local details at multiple scales, balance with global contextual semantic information, and thus effectively retain the fine-grained features of the color of diseased trees and the perception ability of target texture structures.

#### 3.4.2 Cross-scale relative positional encoding and multi-head output

**Figure 5:**
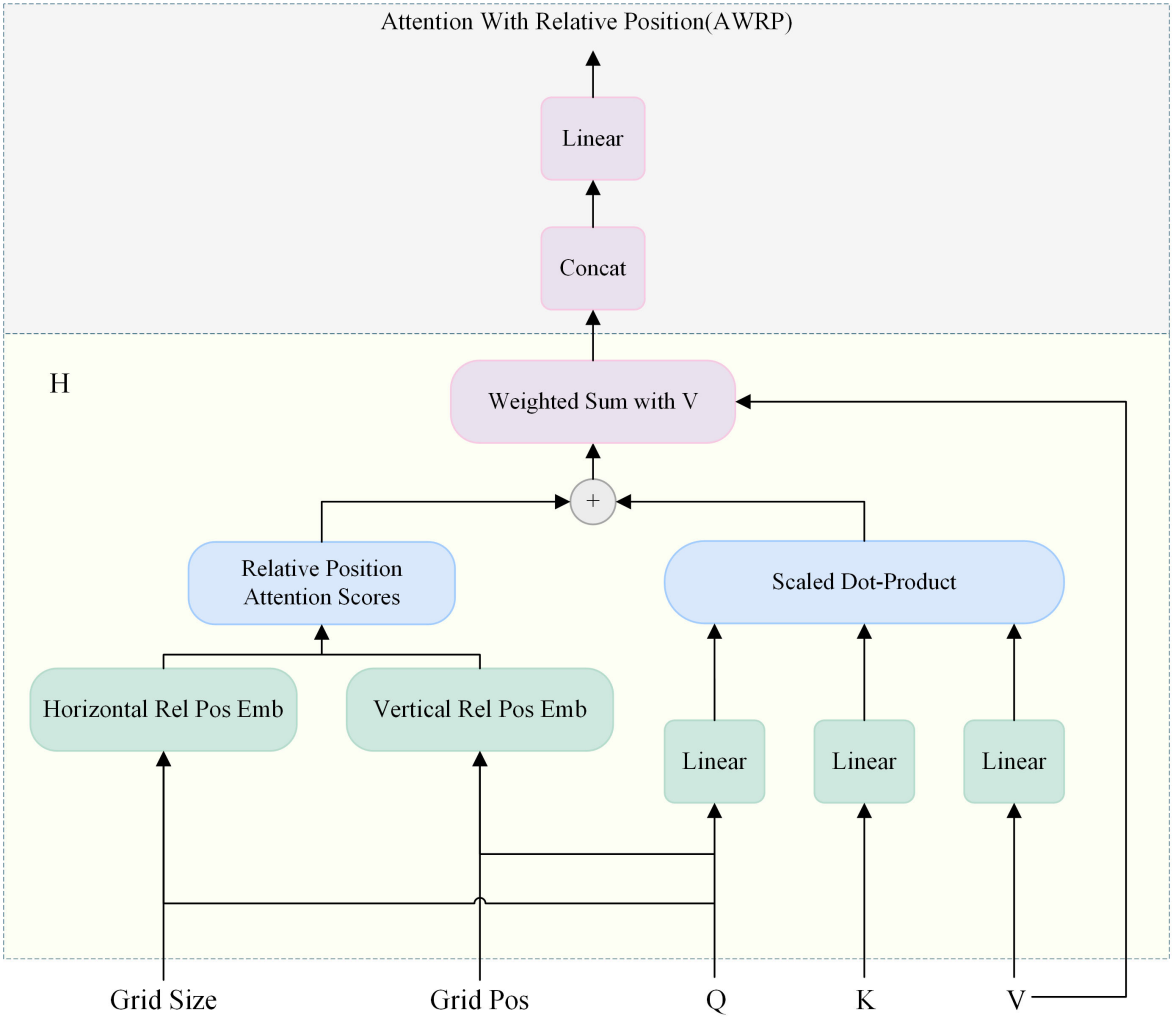
Detailed structure of the proposed AWRP module.

Traditional ViT models embed absolute positional information for global self-attention (Liu et al., 2021). This approach reduces robustness to changes in object scale and spatial layout, making the model overly dependent on content correlation. When applied to pine forest detection, crown occlusion and changes in UAV flight angles can alter how disease features appear. For example, a diseased tree may show clear initial lesions from one angle but appear normal from another, or lighting differences caused by angle changes can mask key visual cues. These physical variations limit the model’s ability to locate lesions accurately. To address sensitivity to texture and structural changes across frames, this study introduces a two-dimensional additive geometric bias into the attention module, improving its ability to remember and focus on disease features. This enhanced attention mechanism, named AWRP, strengthens feature consistency and spatial awareness under varying UAV observation angles, as shown in **Figure 5**.

In this paper, learnable position embedding vectors are integrated into the attention weight generation process, and embedded vector sets R for relative positions are introduced respectively in the two dimensions of vertical Δz and horizontal Δw. A correlation model for capturing adjacent features in remote sensing images is established in the X and Y axis directions of target features, and the model is extended to image features at non-perpendicular angles through additive geometric bias, forming a spatial projection on the two-dimensional plane. As the mapping of shooting angle and position changes, the model can effectively learn the correlation information of marked lesion features in plane and spatial structure. Subsequently, the spatial projection converts the patch feature *Z* into *Q, K*, and *V* vectors. Currently, the two-dimensional relative vectors Δz and Δw of the patch are weighted with the multi-head output module in two directions, imitating the form of multi-head attention, and outputting the weight values of relative positional encoding attention at different scales. The calculation formula is as follows:

(17) $${\text{L}}_{\text{ij}}^{\left(\text{a}\right)}=\frac{\langle {\text{Q}}_{\text{i}}^{\left(\text{a}\right)},\;{\text{K}}_{\text{j}}^{\left(\text{a}\right)}\rangle }{\sqrt{\text{d}}}+\frac{\langle {\text{Q}}_{\text{i}}^{\left(\text{a}\right)},{\text{R}}_{0}^{\left(\text{w}\right)}\;|\Delta \text{w}\;\rangle }{\sqrt{\text{d}}}+\frac{\langle {\text{Q}}_{\text{i}}^{\left(\text{a}\right)}{\text{,R}}_{0}^{\text{(z)}}|\Delta \text{z}\rangle }{\sqrt{\text{d}}}$$

In Equation 17, a denotes the attention head, d represents the dimension, and ij is the coordinate grid. The finally output relative positional encoding attention weight $L_{ij}^{\left(a\right)}$ is coupled with the local masks learned at various scales *via* softmax and then processed in the MLPC (MLP with Conv). Compared with the original MLP, this structure effectively enhances the convolutional spatial processing of patch features, strengthens the fusion of local features and global information, and finally obtains the output feature sequence information.

### 3.5 Attention-guided feature fusion module and output

After processing by the multi-CNN layers in the UNet encoder, local fine-grained features and boundary information are well captured, but the global receptive field remains limited. To address this, a multi-scale feature fusion module is designed to strengthen global perception. This module fuses the global feature sequence from the ViT branch with the downsampling features in the encoder, using a bidirectional attention-gating mechanism layer by layer. The fused features are then passed to the upsampling path to enhance feature perception. Structurally, the module first rearranges the patch encoding with relative positional information into 2D features, which are then aligned and fused with the same-scale downsampling outputs *via* bilinear interpolation. Compared with direct concatenation, this alignment-based fusion improves feature interpretability and strengthens the correlation between local and global information. It also reduces boundary misalignment and geometric distortion during cross-scale fusion. During fusion, a bidirectional gating channel is used. Features of different scales and receptive fields are activated by a Sigmoid function and multiplied element-wise with the original pixels to generate $X^{\prime }$ and ${\hat{T}}^{"}$. These features are then fused through a fully connected layer and refined using depthwise separable convolution and context attention. This process enhances pixel-level feature precision and suppresses redundant attention regions. The computational logic is as follows in Equation 18,

(18) $$\Phi (F)=LCA(DSC_{dil}(RELU(BN(DSC(Concat(X^{\prime }\hat{T^{\prime }}))))))$$

LCA denotes the Lightweight Contextual Attention module. $X^{\prime }$ and ${\hat{T}}^{\prime }$ represent local and global semantics, and Φ(F) is the optimized output feature map. The two branches run independently, and concatenation is applied only at the end. In this way, the usual cross-branch, cross-scale concatenation with $N^{2}$ cost is simplified to a same-dimension concatenation with roughly linear cost. The module aligns global multi-scale features with local details and then applies point-wise enhancement for pixel-level recalibration. As a result, anchor regions are pushed toward likely disease areas. Boundary cues around these anchors are emphasized, and long-range dependencies among features are strengthened, so contextual details become clearer. In practice, this tends to improve sensitivity to small targets and mixed-scale samples, and it helps localize and segment overlapping crowns. Finally, the attention is extended with simple 2D offsets to couple spatial structure and color distribution of diseased trees. By reinforcing correlated information, the model shows better resistance to blur and noise and a lower chance of misrecognition, while keeping the segmentation and localization more stable.

### 3.6 Optimization of hybrid loss function

For remote sensing images of forest areas, scenes are divided into three types based on stand density: dense, regular, and sparse. Diseased tree features are further divided into three stages according to their visual differences. The main goal is to improve segmentation accuracy across these disease stages. To achieve this, a multi-type hybrid loss function is introduced for pixel-level segmentation. It enhances class distinction and reduces inter-class confusion under complex forest backgrounds. Specifically, ArcFace loss is adopted to perform classification prediction on the overall disease distribution degree in forest areas. The classification results are then fed back into the segmentation loss to adaptively adjust coefficients and strengthen feature perception during training. In the model, classification prediction occurs in the downsampling stage, while segmentation prediction is generated at the end of the upsampling stage. Unlike fixed-coefficient methods that separate classification and segmentation, the proposed approach links both through a feedback mechanism that dynamically adjusts global attention weights on pixel semantics. ArcFace loss increases feature separability through a margin-based distance measure related to scene density, making it suited for density-aware classification and assisting segmentation learning.

The hybrid loss function primarily addresses the problem of inter-class similarity among disease features. During feature training, predicted results tend to stabilize only after undergoing various dynamic changes. In the process of feature learning, this study gradually and dynamically adjusts the combination weights of the segmentation loss function based on the semantic information of various disease features circulating in the network. This adjustment aims to find the optimal weight values and balance the impacts of missed detection and false detection. For initial stage symptoms that are prone to false detection, Focal loss is mainly used for category focusing. Moreover, the weight of Focal loss for initial features is adjusted according to different density scenarios output by classification prediction: a higher weight is assigned to initial-stage diseases in sparse scenarios to meet the requirements of small target segmentation, while a lower weight is used in other scenarios.

For the hybrid loss function, let B be the batch size, H and W represent the spatial dimensions of the feature map, and H·W denotes the total number N of spatial positions. Before accurately classifying the current regional density, the model will gradually adjust its category-specific focusing coefficient *γ_n_(x)* until the classification is accurate. This adjustment helps avoid local overfitting or feature omission, accelerates model convergence, and ensures that features in all scenarios can obtain optimal learning parameters. The expression is as follows, where ${\text{W}}_{\text{c}}$ denotes the initial weight of each category and $p_{c}\left(x\right)$ represents the pixel segmentation accuracy in Equation 19,

(19) $$\begin{array}{c} L_{Focal}=-\frac{1}{B\cdot H\cdot W}\sum_{b=1}^{B}\sum_{x=1}^{H\cdot W}\sum_{c=1}^{3}W_{c}\cdot \gamma_{c}\left(x\right)\cdot p_{c}\left(x\right)\cdot log\left(p_{c}\left(x\right)\right) \end{array}$$

Meanwhile, Binary Cross-Entropy (BCE) is introduced as the boundary loss function to enhance the model’s boundary sensitivity under noisy and blurred scenarios. $F=W_{c}\cdot \gamma_{c}\left(x\right)$ is mapped to the boundary distribution characteristics of various features, and the loss values of different categories are adjusted based on this mapping. The expression is as follows in Equations 20, 21, where $B_{c}\left(x\right)$ represents the real mask distribution value and branch correspond to Horizontal (H), Diagonal (D), and Vertical (V) boundaries:

(20) $$\begin{array}{c} BCE\left(p,y\right)=-\left[ylnp+\left(1-y\right)ln\left(1-p\right)\right] \end{array}$$

(21) $$\begin{array}{c} L_{boundary}=\sum_{branch=1}^{3}\sum_{c=1}^{3}\frac{F}{B_{c}}\cdot BCE\left(p_{c}\left(x\right),B_{c}\left(x\right)\right) \end{array}$$

Tversky loss is further introduced to specifically optimize the false detection problem between different disease stages. The false detection penalty weight α is increased in dense areas, and the missed detection penalty weight β is enhanced in sparse areas—this strengthens the model’s attention to the balance between missed detection and false detection. Additionally, contrast loss (Hadsell et al., 2006) is adopted to optimize the distinguishability of easily confused features, thereby increasing true positives ( $TP_{c}$) and reducing false positives for category ( $FP_{c}$) and False Negative ( $FN_{c}$). Its value is calculated as $L_{contrast}$. The expression of Tversky loss is shown below, In Equation 22, $C_{N}$ is the sample size and ϵ is the fixed offset value:

(22) $$\begin{array}{c} \;L_{tversky}=\frac{1}{C_{N}}\sum_{c\in C_{N}}\left(1-\frac{TP_{c}+\epsilon }{TP_{c}+\alpha_{c}\cdot FN_{c}+\beta_{c}\cdot FP_{c}+\epsilon }\right),\;\epsilon \in (0,1) \end{array}$$

The final segmentation loss function is presented as follows in Equation 23. Based on the precision and recall of the classification results themselves, the two weights a and b are dynamically adjusted to balance precision and recall:

(23) $$\;\begin{array}{c} L_{seg}=a\cdot L_{focal}+b\cdot L_{tversky}+0.1\cdot L_{boundary}+0.1\cdot L_{contrast}\;,\;a+b=0.8 \end{array}$$

Prior work supports a main–auxiliary design for segmentation losses. Region objectives are the primary driver because evaluation focuses on region overlap and class imbalance is common strong systems therefore train with region-focused loss functions as the main objective. Abraham and Khan proposed a novel Focal Tversky Loss function (Abraham and Khan, 2019). It transforms the Tversky index using a modulating exponent γ that guides the model to pay greater attention to hard-to-classify samples, small-to-medium-sized samples and samples with less distinct features during training. This approach achieves a better balance between precision and recall, ultimately enhancing the model’s capability in feature extraction and segmentation for specific scenarios. It follows that for segmenting features with diverse shapes and appearances, a loss function that combines the hard-example focus of Focal Loss with the sensitive awareness of segmentation overlap from Tversky Loss *via* a controllable trade-off has proven to be an effective solution with considerable optimization potential.

Other hybrid loss designs such as Combo Loss further show that a main loss plus small auxiliaries outperforms single losses while keeping optimization stable (Taghanaki et al., 2019). To further stabilize the loss function in complex scenes with a large imbalance between foreground and background pixels, we use a boundary loss and a contrast loss at the pixel level as auxiliary terms for Focal loss and Tversky loss, which improves fine segmentation of diseased regions and helps separate confusing features. In terms of combination methods, these terms work best with modest coefficients (Tang et al., 2024). In our task, the model trains mainly with a region focused loss made of Focal and Tversky, and this drives convergence. To avoid mixing objectives and to keep training stable, we will give the auxiliary losses small weights. Under this setting, 80 percent of the total weight are assigned to Focal and Tversky (a+b=0.8), and Boundary and Contrast loss weights are set to 0.1 each. It aligns training with region-overlap metrics and lets the auxiliaries correct edge errors and reduce class confusion without dominating the objective. We let the ArcFace scene-density classifier output $p_{\text{dense}},p_{\text{regular}},p_{\text{sparse}}$. When $p_{\text{regular}}<\;max(p_{\text{sparse}},\;p_{\text{dense}}$*)*, we define the dense–sparse ratio $r=\frac{p_{\text{dense}}}{p_{\text{dense}}+p_{\text{sparse}}}$ and the confidence $c=max\left(p_{\text{dense}},p_{\text{sparse}}\right)$. Use a confidence-shaped mapping

(24) $$\;F\left(r,c\right)=\frac{r^{\;1+\alpha \left(c-0.5\right)}}{\;r^{1+\alpha \left(c-0.5\right)}+{\left(1-r\right)}^{1+\alpha \left(c-0.5\right)}\;},\alpha >0$$

and set the region weights.

(25)  b=0.8 F(r,c),a=0.8−b

In Equations 24, 25, if $p_{regular}>max(p_{sparse},\;p_{dense}$*)*, we do not change the weight. Thus the model raises a (stronger Focal) to emphasize rare, small initial-stage lesions against large backgrounds in sparse scenes, and raises b (stronger Tversky) to control false negatives and overlap errors in crowded crowns in dense scenes. This feedback only reallocates a  and b  but does not change inference.

## 4 Experiment results

### 4.1 Experimental setup

In the experiment, we first initialized the parameters of the MSCF-LUNet network, and then conducted training on the multi-stage PWD dataset. Experiments ran on a single NVIDIA V100 (32 GB) GPU using AdamW with a 10 epochs rmup, an initial learning rate of $2\times 10^{-5}$, and weight decay of $1\times 10^{-6}$. During the training process, we recorded the convergence points of each model at their peak performance, and evaluated their effectiveness using datasets with different interference intensities and interference ratios. In this study, precision, recall, IoU (Intersection over Union), and PA (Pixel Accuracy) were adopted as the model evaluation metrics.

PA refers to the proportion of correctly classified pixels among all pixels. Precision denotes the proportion of pixels predicted as a specific class by the model that actually belong to that class. Recall represents the proportion of pixels that are actually of a specific class and correctly predicted as that class by the model. IoU of MSCF-LUNet reflects the overlap between the predicted region and the actual region, which indirectly indicates its localization ability. The mean values of these indicators are denoted as mPA (mean Pixel Accuracy), mP (mean Precision), mR (mean Recall), and mIoU (mean IoU). In addition, this study introduces Parameters (M), FLOPs (G), and Weight (MB) to represent the lightweight performance, which respectively denote the structural complexity of the model, the computational complexity of the model, and the weight size of the model. The main task of this model is the localization and segmentation of multi-stage disease features. The calculation formula for these evaluation indicators is as follows in Equations 26–29:

(26) $$\;mPA=\frac{1}{K}\sum_{i=1}^{K}\frac{TP_{i}+TN_{i}}{TP_{i}+TN_{i}+FP_{i}+FN_{i}}$$

(27) $$mP=\frac{1}{K}\sum_{i=1}^{K}\frac{TP_{i}}{TP_{i}+FP_{i}}$$

(28) $$\;mR=\frac{1}{K}\sum_{i=1}^{K}\frac{TP_{i}}{TP_{i}+FN_{i}}\;$$

(29) $$mIOU=\frac{1}{K}\sum_{i=1}^{K}\frac{TP_{i}}{TP_{i}+FP_{i}+\text{F}N_{i}}$$

$TP_{i}$ denotes pixels correctly predicted as class i, $FP_{i}$ denotes pixels that are not of class i but predicted as class i, $TN_{i}$ (True Negative) denotes pixels that do not belong to class i and are not predicted as class i, $FN_{i}$ denotes pixels that actually belong to class i but are not predicted as class i, K  is the number of classes considered.

### 4.2 Ablation experiments

We evaluated the impact of each module in the ablation study, including improved DSC, LCA, MSF, and LUNet, on the baseline UNet through a series of experiments. The backbone convolutions were replaced with depthwise separable convolution to form a DoubleConv block, and LCA attention was added at the downsampling and feature fusion stages. A multi-hybrid loss function was used as the default training setup. Training and testing were carried out under random scene conditions. The segmentation results were combined with density classification outputs from the downsampling stage, and the final predictions were generated at the end of the upsampling stage. Detailed experimental results are shown in **Table 3**.

**Table 3:** The main segmentation and auxiliary classification results of the model.

MSCF-LUNet	Scene	P (%)	R (%)
classification	Scattered	90.13	91.24
	Regular	92.83	92.14
	Dense	92.47	91.89
segmentation	Initial	88.45	90.02
	Intermediate	89.56	93.13
	Advanced	90.67	93.24

After pruning and compressing UNet, the model showed decreases of 4.44%, 4.94%, 5.87%, and 7.18% in mP, mR, mIoU, and mPA respectively compared with the original model, but significantly saved the amount of computation and parameters. After introducing each module, the model finally achieved 89.56%, 92.13%, 88.92%, and 96.54% in the above metrics, which were 5.00%, 6.76%, 3.78%, and 1.72% higher than the standard model respectively. At this time, the model parameter count was 23.28M, which was 7.97M lower than the standard model; the computation amount was 64.17G, which was 334.96G lower; and the final model weight reached 89.16MB, which was 30.10MB lower than the standard model. The performance improvement was particularly significant when the improved MSF mechanism was introduced. This indicates that after introducing the MSF mechanism, the local features, under the enhancement of global semantic information from multi-scale hybrid patch embedding and multi-scale relative positional encoding, effectively improved the model’s ability to locate easily confused features in the initial and intermediate stages under the forest background, as well as the pixel-level perception intensity of the overall features, thereby optimizing performance. After further combining LCA and DSC, the results of the overall performance improvement also confirmed that the model’s ability to jointly perceive local information and the global environment is the key to improving feature classification accuracy and overall as well as boundary segmentation accuracy. The segmentation results of MSCF-LUNet for the multi-stage disease features and the classification prediction results of various scenarios are shown in **Table 2**.

The integration of environmental information and feature information further enhanced the model’s ability to perceive easily confused disease features in the initial and intermediate stages. This not only achieved significant progress in the refined perception and segmentation capabilities of various disease features, but also further reduced the missegmentation rate of natural features on other tree species.

### 4.3 Comparison experiment

To verify the effectiveness of the model proposed in this paper and the rationality of the global-local perception mechanism, different backbones were introduced for comparative verification under the same environment and parameters. These backbones include ResNet50, ResNet101 (He et al., 2016), PSPNet (Zhao et al., 2017), FCN (Long et al., 2015), MobileNetV4 (Qin et al., 2024), YOLOv8-seg (Varghese and M., 2024), YOLOv11-seg (Khanam and Hussain, 2024), DeepLabv3+ (Chen et al., 2018), Swin-Unet (Cao et al., 2021), and SegFormer (Xie et al., 2021) Each comparative model has its own advantages and characteristics: some are traditional and popular neural networks, while others are segmentation models that have achieved State-of-the-Art (SOTA) on multiple general datasets in recent years. A performance comparison was conducted between these models, the standard UNet, and MSCF-LUNet on the multi-stage pine pest and disease dataset, with the results shown in **Table 4**. Among them, Swin-Unet, SegFormer, and DeepLabv3+ResNet exhibit relatively outstanding performance. The multi-scale nature of their integrated Transformer and Atrous Spatial Pyramid Pooling (ASPP) endows them with strong anti-interference capabilities; however, limited by the inherent design constraints of the models themselves, their complexity is relatively high.

**Table 4:** Results of mainstream algorithm comparison experiments.

Method	mP (%)	mR (%)	mIoU (%)	mPA (%)	Parameters (M)	FLOPs (G)	Weight (MB)
ResNet50	69.68	71.37	61.80	86.97	25.51	38.34	90.17
ResNet101	70.17	72.88	65.50	92.43	44.02	71.52	109.65
FCN	61.15	53.30	57.80	88.25	134.04	380.33	134.54
PSPNet	65.63	61.22	65.38	79.62	50.17	350.21	101.68
MobileNetV4	51.24	50.42	54.25	74.04	**4.37**	**10.42**	**20.10**
YOLOv8n-seg	65.80	61.20	60.50	85.32	11.15	28.66	48.69
YOLOv11n-seg	68.82	65.14	64.72	85.68	10.93	27.44	45.34
UNet	84.56	85.37	85.14	94.82	31.25	399.13	119.65
DeepLabv3+ResNet	85.17	84.42	84.29	95.24	55.02	520.16	141.23
SegFormer	88.13	89.59	**89.54**	96.12	51.32	443.75	123.41
Swin-Unet	87.87	90.04	87.12	95.67	121.71	788.32	164.47
MSCF-LUNet	**89.56**	**92.13**	88.92	**96.54**	23.28	64.17	89.16

The bold values represent optimal value.

Most traditional networks stack only convolutional layers. They focus on local patterns and fail to integrate scene context. Our analysis shows that blindly deepening convolutions is not suitable for stage detection of pests and diseases from remote sensing images. Too much weight on local cues ignores environmental and spatial effects and increases overfitting. Models with stronger global perception separate background from targets and also separate similar targets from each other, which raises segmentation performance. A refined fusion of global semantics with local detail is therefore better for scenes with many small targets and easily confused features. On our benchmarks, MSCF-LUNet outperforms most baselines across metrics. In complex forest scenes, its mIoU is 0.62% lower than SegFormer, but its mP, mR, and mPA exceed the second-best methods by 1.43%, 2.09%, and 0.42%, respectively. At the same time, parameters and FLOPs are kept low, giving a clear lightweight benefit without hurting core accuracy.

**Figure 6:**
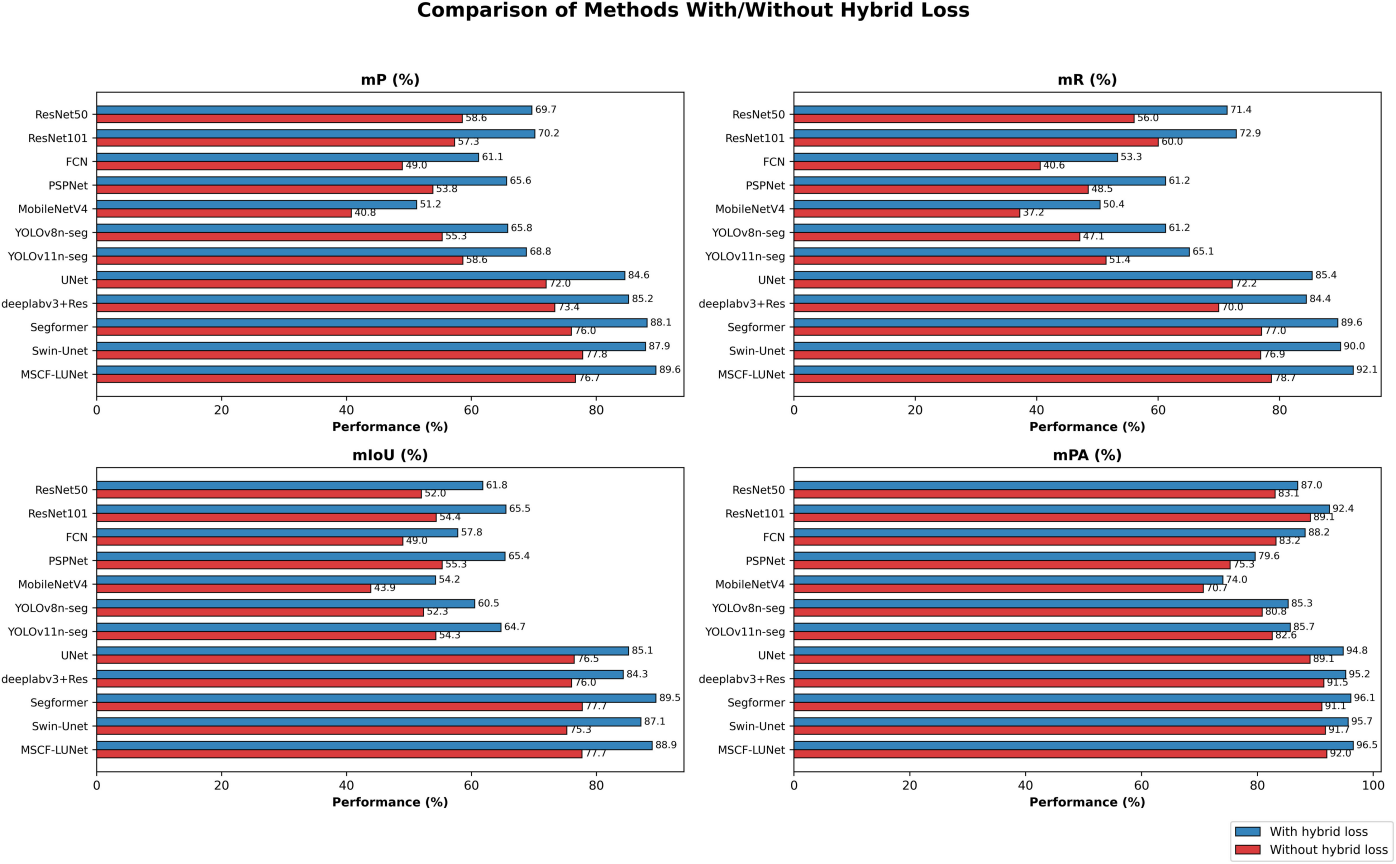
Performance comparison of various models with/without hybrid loss function strategy in this article.

To further demonstrate the performance improvement brought by the hybrid loss function in multi-stage segmentation of PWD, we conducted comparative experiments on all models with and without the hybrid loss. When the hybrid loss function was not applied, the models were trained using the standard Focal Loss. The experimental results are shown in **Figure 6**. It can be observed that, under complex environments, the dynamically coordinated hybrid loss function enables the model to more effectively learn feature details, boundary information, and environmental distributions. As a result, the model is able to capture richer and more effective details, significantly enhancing the generalization ability of existing segmentation models in complex environments and reducing the risk of overfitting. This demonstrates the scientific validity of the proposed hybrid loss function in PWD segmentation tasks.

To further probe and verify the mechanism and characteristics of the hybrid loss, we conducted an ablation study. **Table 5** lists the evaluation metrics, mR and mIoU, and the corresponding results. We first trained with a single Focal loss as the baseline. For completeness, we also evaluated a single Tversky loss under the same setting. We then fused Focal and Tversky without auxiliaries, assigning equal weights of 0.5 and 0.5. Next, we added one auxiliary at a time, namely Boundary or pixel-wise Contrastive, while keeping the region terms dominant with a combined weight of 0.8 and the auxiliary at 0.2. When Focal and Tversky loss were combined, ArcFace was included as a margin-based feedback mechanism control that regulates effective weight allocation and strengthens feature discrimination, which further improves segmentation performance. Finally, we enabled both auxiliaries together, with the region terms fixed at 0.8 and each auxiliary at 0.1. The results show a consistent trend. Either single region loss improves the baseline, and the fused Focal–Tversky setup is stronger than either alone. A single auxiliary brings limited gains and mainly corrects one specific type of confusion, and its performance improvement may not occur synchronously across all metrics. In contrast, the cooperative configuration yields the largest and most stable improvements in both mR and mIoU. This indicates that the dominant performance gains in MSCF-LUNet come from the synergy among the loss components rather than any single one. It also shows that the improvement is not due to added loss function complexity but to the flexible use of complementary feature advantages across different functions.

**Table 5:** Hybrid loss function ablation experiment based on MSCF-LUNet.

Focal	Tversky	BCE	Contrast	ArcFace	mR (%)	mIoU (%)
✔	—	—	—	—	78.7	77.7
—	✔	—	—	—	78.4	78.4
✔	—	✔	—	—	80.4	81.3
✔	—	—	✔	—	80.3	81.1
—	✔	✔	—	—	79.8	80.2
—	✔	—	✔	—	80.1	82.4
✔	✔	—	—	—	85.3	83.6
✔	✔	—	—	✔	88.4	85.5
✔	✔	✔	—	—	86.2	84.9
✔	✔	—	✔	—	86.1	85.2
✔	✔	✔	—	✔	89.2	86.4
✔	✔	—	✔	✔	88.7	87.1
✔	✔	✔	✔	—	89.4	86.8
✔	✔	✔	✔	✔	**92.1**	**88.9**

The bold values represent optimal value.

### 4.4 Analysis of visualization

**Figure 7:**
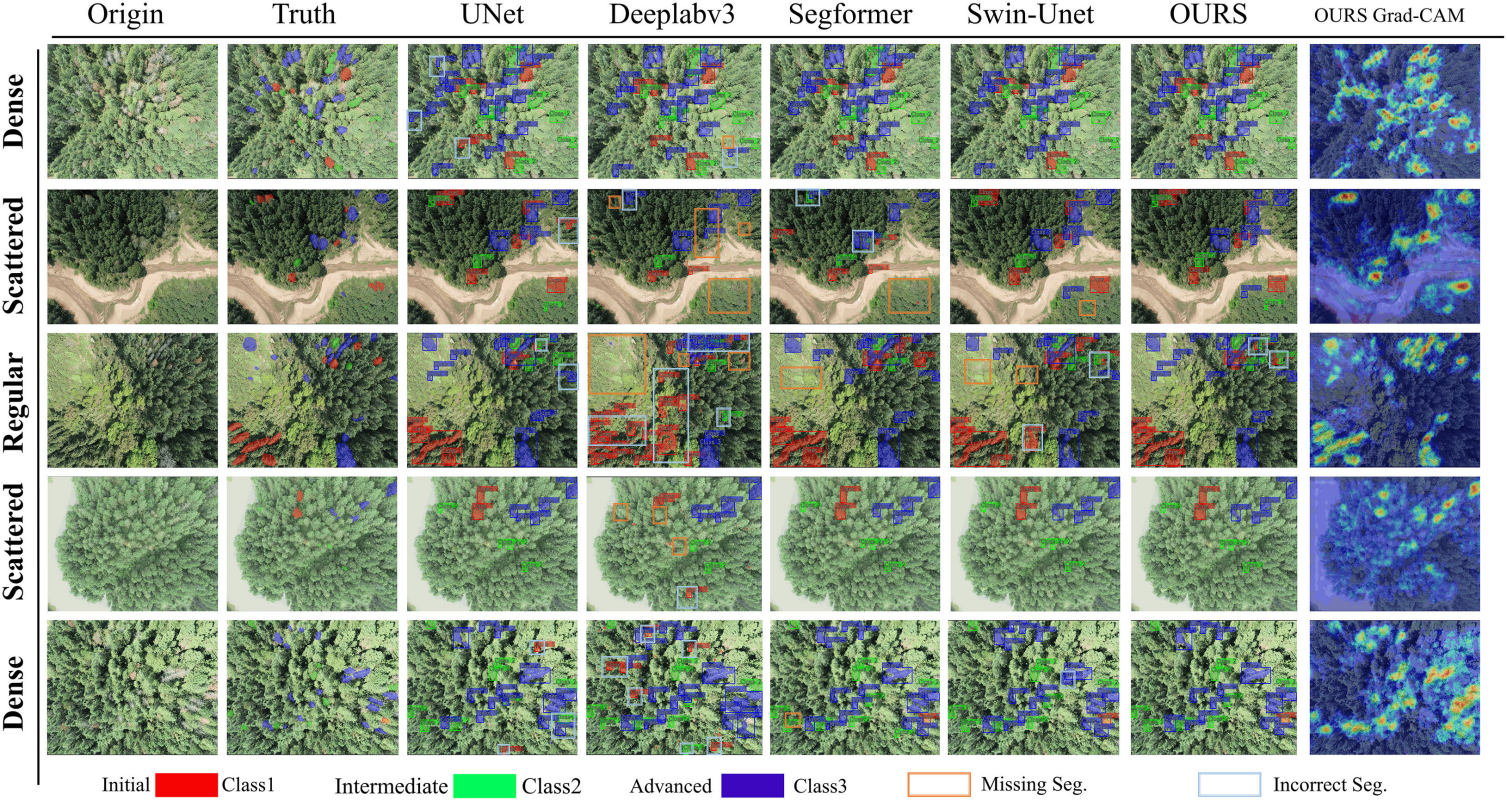
Comparison experiment of mainstream model visualization. The three-stage diseases are represented by red (Initial Class1), green (Intermediate Class2), and blue (Advanced Class3); the orange box indicates missed segmentation, and the light blue box indicates false segmentation.

To balance rapid response and efficiency, we used RGB remote-sensing images for training and validation. We selected five UAV scenes with distinct spatial patterns from the experimental forest and built a visualization dataset from these images. The areas include dense stands, river margins, depressions, and forestland with embedded sparse patches, creating diverse backgrounds and disease distributions. Experiments show that MSCF-LUNet remains robust across these settings and delivers accurate segmentation under common sources of interference, as shown in **Figure 7**.

Compared with standard UNet and other models, MSCF-LUNet performs better in complex scenes with multiple disease features. It captures target shape more accurately, identifies stage cues more reliably, and lowers the false-positive rate from look-alike species and sparse ground patches. By filtering out similar but incorrect patterns, the model achieves accurate segmentation and recognition of diseased trees. Its stronger global perception increases sensitivity to environmental context and enables finer local processing. **Figure 7** also shows that the model can effectively perceive and segment imperceptible small target features, while mitigating the problem of missing external structure and boundary information during feature segmentation, leading to more precise pixel positioning. The efficient relative position attention mechanism not only significantly optimizes the perception capability of overlapping targets but also further improves the segmentation precision of overlapping samples.

The interpretability of MSCF-LUNet was further improved using the Gradient-weighted Class Activation Mapping (Grad-CAM) method. Grad-CAM produces a coarse heatmap by weighting the gradients of the target class with the outputs of a chosen convolutional layer, showing which regions of the image the model focuses on. In the heatmap, colors from red to light yellow represent strong attention, while blue areas indicate irrelevant or noisy regions. Analysis of the fusion layer and segmentation maps shows that although some missed detections remain in heavily interfered scenes, the model maintains strong focus on correct targets in most cases and demonstrates adaptive resistance to interference.

**Figure 8:**
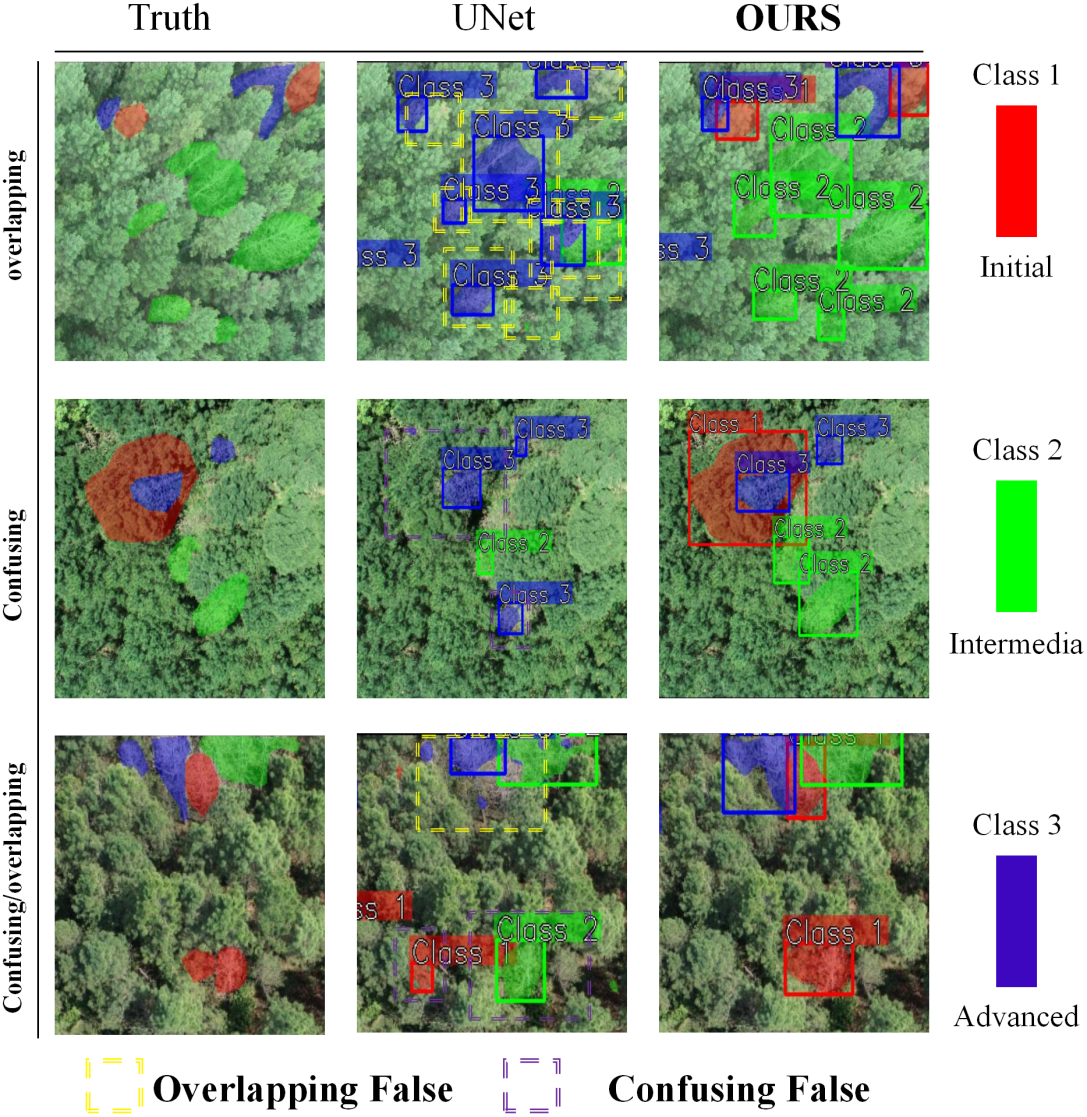
Comparison of visualization effects and detailed analysis of segmentation errors in various complex environments before and after model improvement. Yellow indicates errors caused by overlap, purple indicates errors caused by feature confusion, and the rest are due to the model’s own perceptual capabilities.

To clearly compare MSCF-LUNet with UNet, segmentation visualizations in complex forest scenes containing overlapping and confusing features are shown in **Figure 8**. Three close-up regions were selected, representing overlapping features, confusing features, and their combination. Results show that UNet often misclassifies intermediate-stage trees with sparse crowns as advanced cases and produces uneven segmentation. Under occlusion and heavy overlap, UNet misses many targets and achieves low pixel precision. After introducing the MSF mechanism, MSCF-LUNet effectively reduces missed and false detections and produces smoother, more accurate segmentation. This confirms that the MSF mechanism is key to improving the detection of multi-stage diseased trees in complex environments. By combining environmental context and multi-scale features, the model focuses better on hard samples, extracts more useful cues, and reduces the risk of overfitting.

**Figure 9:**
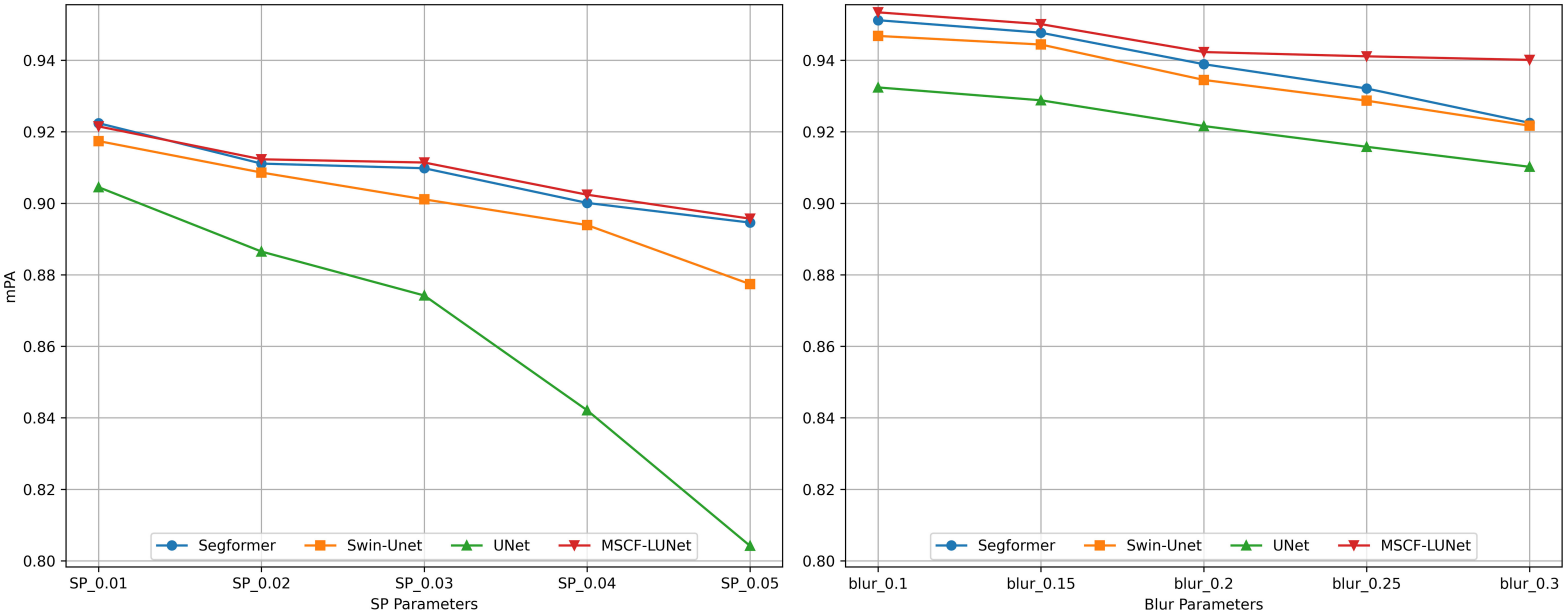
Comparative experiment of different interference methods. The left image shows the interference gradient of salt-and-pepper noise, and the right image shows the interference gradient of dynamic blur.

In addition, UNet, SegFormer, Swin-Unet, and MSCF-LUNet were selected to simulate external interference during remote sensing image segmentation under different gradients of salt-and-pepper noise and motion blur, so as to further verify the anti-interference robustness and scientific validity of the models. **Figure 9**. shows the changes in the models’ mPA after introducing 1%–5% salt-and-pepper noise pixels and motion blur with an intensity of 0.1–0.3. By analyzing mPA, we can obtain the accuracy of the model in correctly recognizing and segmenting instance features under interference, which reflects the robustness of the model. Salt-and-pepper noise introduces black and white pixels, which are similar to the color features of advanced-stage diseases.

Among the four models tested, MSCF-LUNet shows the smallest drop in accuracy when salt-and-pepper noise is added. Even under the strongest noise interference, it still keeps a high mPA. This shows that the model can use global context to capture the spatial structure of disease features and maintain good robustness against this kind of noise. Motion blur, on the other hand, weakens spatial structure and increases confusion with similar-looking trees. Across different blur levels, MSCF-LUNet continues to perform well, and its accuracy drops much less than that of the other models. The result suggests that the model’s multi-scale local perception helps recover missing structure and maintain stable performance under motion blur.

**Figure 10:**
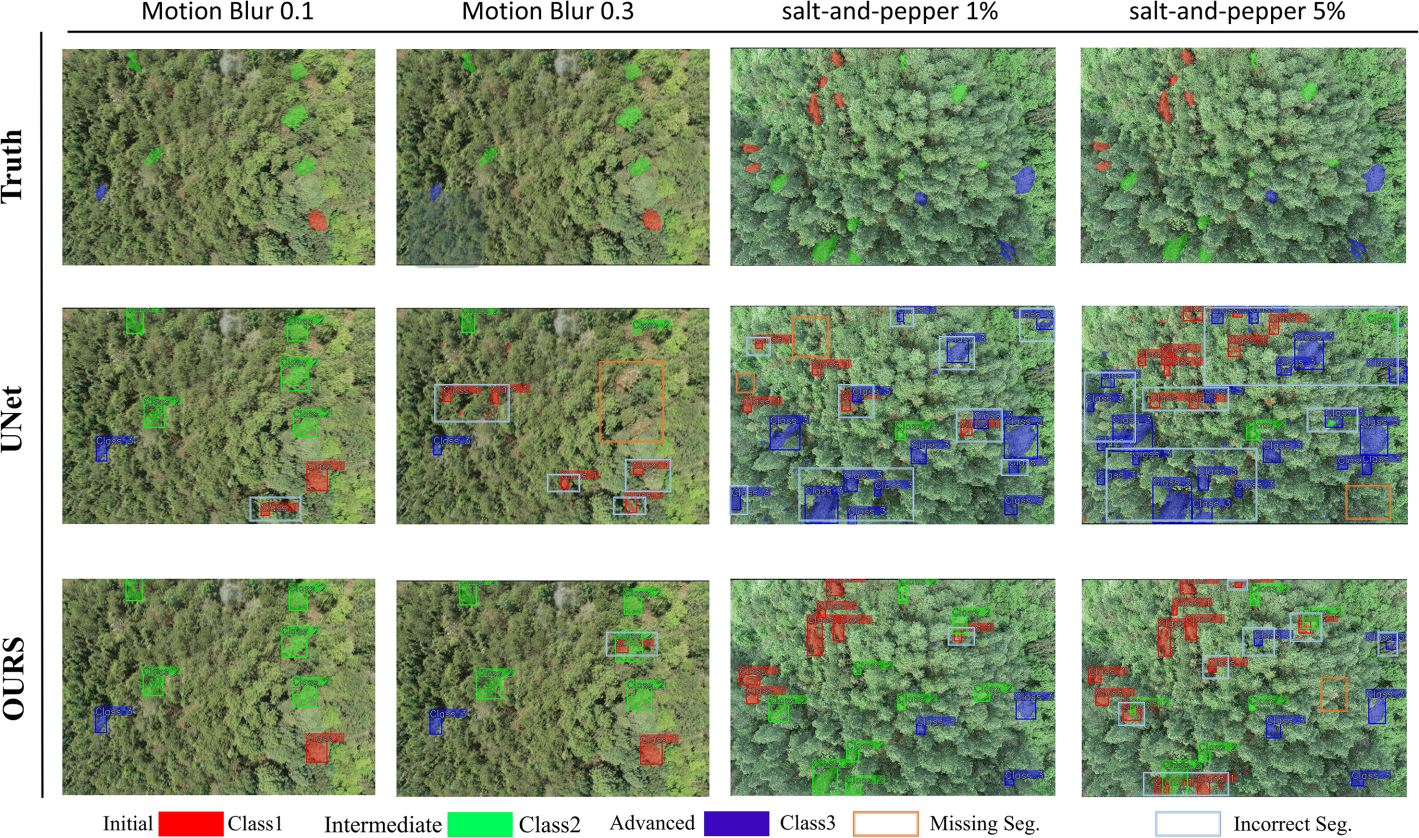
Anti-interference test results. The left half shows the segmentation under motion blur, while the right half shows the segmentation under salt-and-pepper noise, both at two intensity levels.

For the other three models, accuracy declines quickly as the level or area of interference grows. Even compared with models that also include global perception, MSCF-LUNet’s mixed-scale design and relative-position awareness use both context and fine details more effectively, without adding extra heavy modules. To show this visually, we compared samples with the lowest and highest interference levels in both settings. **Figure 10** shows that UNet often produces false detections under light motion blur, mistaking similar features for initial-stage symptoms. As the blur increases, the problem worsens and true targets are missed. In contrast, MSCF-LUNet keeps stable segmentation, with only small overlaps even in strong blur. Under salt-and-pepper noise, UNet tends to misclassify intermediate-stage features as advanced ones and ignore confusing regions. But MSCF-LUNet maintains high accuracy, with only a few minor errors, and avoids large-scale misjudgment. Its resistance to interference and confusion is clearly stronger than that of the baseline models.

## 5 Discussion

While RGB-based remote-sensing segmentation datasets are easy to collect and useful for quickly verifying model performance, they still face limitations in terms of high accuracy and reliability. When constructing the PWD remote-sensing dataset, the sampling of RGB images based on typical PWD features still carries a certain degree of scene randomness that is hard to avoid. Then some initial-stage cases may still be missed. Even so, across forests with diverse terrain, we still collected many kinds of initial-stage samples covering different visual forms. A multi-step verification with UAV prescreening, expert field checks, and cross review kept stage definitions consistent and avoided mixing features between stages. The labels are reliable for training and generalization, and we found no systematic mislabeling of initial-stage PWD. In future work, we will measure chlorophyll and key branch such as leaf traits from typical initial-stage trees in the lab. These traits will be aligned with hyperspectral indices and RGB color-texture features, then fused for training to build a stronger multimodal dataset.

MSCF-LUNet is not an ultra-lightweight model for micro-edge devices; instead, it is designed for local workstation GPUs or cloud servers. Very compact networks such as MobileNetV4 have 4.37M parameters and 10.42G FLOPs, but achieve only 54.25% mIoU on our dataset. These models are small, but they do not satisfy the accuracy requirements of fine-grained stage segmentation in complex scenes.

Our objective is to design a relatively balanced and lightweight model that preserves high accuracy while removing most of the computation of heavy SOTA segmentation networks. This trade-off is appropriate for scenarios in which UAV images are transmitted to local or cloud GPUs for rapid segmentation. MSCF-LUNet has 23.28M parameters, 64.17G FLOPs, and a model size of 89.16 MB. These values are substantially smaller than those of the segmentation backbones used for comparison: UNet (31.25M/399.13G/119.65 MB), SegFormer (51.32M/443.75G/123.41 MB), and Swin-UNet (121.71M/788.32G/164.47 MB). Thus, MSCF-LUNet reduces computation by approximately 84–92% and parameters by 26–81% relative to these networks, while maintaining high accuracy.

From a deployment perspective, UAV images can be transmitted for processing shortly after acquisition. In such settings, the reduced parameters and FLOPs of MSCF-LUNet directly translate into lower inference time and hardware demand compared with large segmentation backbones, but without the severe accuracy loss observed in ultra-compact models.

In future work, we plan to design more compact feature fusion modules that reuse information more efficiently and reduce redundant computation. The goal is to preserve the stage-level segmentation accuracy demonstrated in this study, while further lowering the model size and computational cost so that real-time semantic segmentation on edge hardware becomes feasible.

## 6 Conclusion

We present a lightweight model for segmenting PWD in complex forest scenes. MSCF-LUNet pairs relative-position attention with a multi-scale patch-embedding path. The fusion pulls useful detail from cluttered backgrounds and also captures scene context, so global semantics are easier to localize. Inside the encoder, context attention tightens information flow between layers. It guides fusion of same-scale local features with outputs from the multi-scale path. Global cues are aligned with local details step by step, and the decoder produces the final high-precision masks. In tests, the model works well for PWD semantic segmentation across many scenarios. It leads on average performance. Under motion blur and salt-and-pepper noise, it still beats mainstream baselines. The takeaway: MSCF-LUNet keeps strong feature analysis and robustness, and it segments and localizes PWD accurately in complex environments.

## Data Availability

The original contributions presented in the study are included in the article/**Supplementary Material**. Further inquiries can be directed to the corresponding authors.

## References

[B1] Abraham N. Khan N. M. (2019). “ A Novel Focal Tversky loss function with Improved Attention U-Net for lesion segmentation,” in 2019 IEEE 16th International Symposium on Biomedical Imaging (ISBI 2019). 683–687. doi: 10.1109/ISBI.2019.8759329

[B2] Cai X. Lai Q. Wang Y. Wang W. Sun Z. Yao Y. (2024). “ Poly kernel inception network for remote sensing detection,” in IEEE/CVF Conference on Computer Vision and Pattern Recognition ( IEEE, Seattle, WA, USA), 27706–27716. doi: 10.1109/CVPR52733.2024.02617

[B3] Cao H. Wang Y. Chen J. Jiang D. Zhang X. Tian Q. . (2021). “ Swin-unet: Unet-like pure transformer for medical image segmentation,” in European Conference on Computer Vision ( Springer Nature Switzerland, Cham), 205–218. doi: 10.1007/978-3-031-25066-8_9

[B4] Chen J. Lu Y. Yu Q. Luo X. Adeli E. Wang Y. . (2021). TransUNet: Transformers Make Strong Encoders for Medical Image Segmentation ( arXiv). Available online at: https://arxiv.org/abs/2102.04306 (Accessed August 15, 2025).

[B5] Chen L. C. Zhu Y. Papandreou G. Schroff F. Adam H. (2018). “ Encoder-decoder with atrous separable convolution for semantic image segmentation,” in European Conference on Computer Vision. doi: 10.1007/978-3-030-01234-2_49

[B6] Deng J. Guo J. Yang J. Xue N. Kotsia I. Zafeiriou S. (2022). ArcFace: additive angular margin loss for deep face recognition. IEEE Trans. Pattern Anal. Mach. Intell. 44, 5962–5979. doi: 10.1109/TPAMI.2021.3087709, PMID: 34106845

[B7] Deng X. Tong Z. Lan Y. Huang Z. (2020). Detection and location of dead trees with pine wilt disease based on deep learning and UAV remote sensing. AgriEngineering. 2, 294–307. doi: 10.3390/agriengineering2020019

[B8] Dong X. Zhang L. Xu C. Miao Q. Yao J. Liu F. . (2024). Detection of pine wilt disease infected pine trees using YOLOv5 optimized by attention mechanisms and loss functions. Ecol. Indic. 168, 112764. doi: 10.1016/j.ecolind.2024.112764

[B9] Dosovitskiy A. Beyer L. Kolesnikov A. Weissenborn D. Zhai X. Unterthiner T. . (2021). “ An image is worth 16x16 words: Transformers for image recognition at scale,” in International Conference on Learning Representations. doi: 10.48550/arXiv.2010.11929

[B10] Du Z. Wu S. Wen Q. Zheng X. Lin S. Wu D. (2024). Pine wilt disease detection algorithm based on improved YOLOv5. Front. Plant Sci. 15. doi: 10.3389/fpls.2024.1302361, PMID: 38699534 PMC11063304

[B11] Hadsell R. Chopra S. LeCun Y. (2006). “ Dimensionality reduction by learning an invariant mapping,” in Proceedings of the 2006 IEEE Conference on Computer Vision and Pattern Recognition ( IEEE, Piscataway, NJ, USA), 1735–1742. doi: 10.1109/CVPR.2006.100

[B12] He K. Zhang X. Ren S. Sun J. (2016). “ Deep residual learning for image recognition,” in IEEE Conference on Computer Vision and Pattern Recognition ( IEEE, Las Vegas, NV, USA), 770–778. doi: 10.1109/CVPR.2016.90

[B13] Iordache M. D. Mantas V. Baltazar E. Pauly K. Lewyckyj N. (2020). A machine learning approach to detecting pine wilt disease using airborne spectral imagery. Remote Sens. 12, 2280. doi: 10.3390/rs12142280

[B14] Jung Y. Byun S. Kim B. Ul Amin S. Seo S. (2024). Harnessing synthetic data for enhanced detection of Pine Wilt Disease: An image classification approach. Comput. Electron. Agric. 218, 108690. doi: 10.1016/j.compag.2024.108690

[B15] Kervadec H. Bouchtiba J. Desrosiers C. Granger E. Dolz J. Ayed I. B. (2021). Boundary loss for highly unbalanced segmentation. Med. Image Anal. 67, 101851. doi: 10.1016/j.media.2020.101851, PMID: 33080507

[B16] Khanam R. Hussain M. (2024). YOLOv11: An Overview of the Key Architectural Enhancements ( arXiv). Available online at: https://arxiv.org/abs/2410.17725 (Accessed August 15, 2025).

[B17] Li H. Zhou G. Liu J. Zhang H. (2011a). “ Study on pine wilt disease and its control situation,” in Applied Mechanics and Materials. Ed. Luo Q. ( Trans Tech Publications Ltd, Switzerland), 567–572.

[B18] Lin M. Chen Q. Yan S. (2014). “ Network in network,” in International Conference on Learning Representations. doi: 10.48550/arXiv.1312.4400

[B19] Lin T.-Y. Goyal P. Girshick R. He K. Dollár P. (2020). “ Focal loss for dense object detection,” in IEEE Transactions on Pattern Analysis and Machine Intelligence. 318–327. doi: 10.1109/TPAMI.2018.2858826, PMID: 30040631

[B20] Liu H. Li W. Jia W. Sun H. Zhang M. Song L. (2024). Clusterformer for pine tree disease identification based on UAV remote sensing image segmentation. IEEE Trans. Geosci. Remote Sens. 62, 1–15. doi: 10.1109/TGRS.2024.3362877

[B21] Liu Z. Lin Y. Cao Y. Hu H. Wei Y. Zhang Z. . (2021). “ Swin Transformer: Hierarchical vision transformer using shifted windows,” in IEEE/CVF International Conference on Computer Vision ( IEEE, Montreal, QC, Canada), 9992–10002. doi: 10.1109/ICCV48922.2021.00986

[B22] Long J. Shelhamer E. Darrell T. (2015). “ Fully convolutional networks for semantic segmentation,” in Proceedings of the 2015 IEEE Conference on Computer Vision and Pattern Recognition ( IEEE, Boston, MA, USA), 3431–3440. doi: 10.1109/CVPR.2015.7298965 27244717

[B23] Mamiya Y. Kiyohara T. (1972). Description of bursaphelenchus lignicolus N. Sp. (Nematoda: aphelenchoididae) from pine wood and histopathology of nematode-infested trees. Nematologica 18, 120–124. doi: 10.1163/187529272X00296

[B24] NFGA (2024). Technical Scheme for Pine Wilt Disease Prevention and Control. 2024 Edition (Beijing, China: Technical Document).

[B25] Ouyang D. He S. Zhang G. Luo M. Guo H. Zhan J. (2023). “ Efficient multi-scale attention module with cross-spatial learning,” in Proceedings of the 2023 IEEE International Conference on Acoustics, Speech and Signal Processing ( IEEE, Rhodes Island, Greece), 1–5. doi: 10.1109/ICASSP49357.2023.10096516

[B26] Pan J. Ye X. Shao F. Liu G. Liu J. Wang Y. (2024). Impacts of pine species, infection response, and data type on the detection of Bursaphelenchus xylophilus using close-range hyperspectral remote sensing. Remote Sens. Environ. 315, 114468. doi: 10.1016/j.rse.2024.114468

[B27] Qin D. Leichner C. Delakis M. Fornoni M. Luo S. Yang F. . (2024). MobileNetV4 - Universal models for the mobile ecosystem. Eur. Conf. Comput. Vision. 15098. doi: 10.1007/978-3-031-73661-2_5

[B28] Ronneberger O. Fischer P. Brox T. (2015). “ U-net: convolutional networks for biomedical image segmentation,” in Proceedings of Medical Image Computing and Computer-Assisted Intervention – MICCAI 2015. Eds. Navab N. Hornegger J. Wells W. M. Frangi A. F. ( Springer Int. Publ, Cham), 234–241. doi: 10.1007/978-3-31924574-4_28

[B29] Shen J. Xu Q. Gao M. Ning J. Jiang X. Gao M. (2024). Aerial image segmentation of nematode-affected pine trees with U-net convolutional neural network. Appl. Sci. 14, 5087. doi: 10.3390/app14125087

[B30] TaGhanaki S. A. Zheng Y. Kevin Zhou S. Georgescu B. Sharma P. Xu D. . (2019). Combo loss: Handling input and output imbalance in multi-organ segmentation. Computerized Med. Imaging Graphics 75, 24–33. doi: 10.1016/j.compmedimag.2019.04.005, PMID: 31129477

[B31] Tang H. Zhao G. Gao J. Qian X. (2024). Personalized representation with contrastive loss for recommendation systems. IEEE Trans. Multimedia 26, 2419–2429. doi: 10.1109/TMM.2023.3295740

[B32] Tudor C. Constandache C. Dinca L. Murariu G. Badea N. O. Tudose N. C. . (2025). Pine afforestation on degraded lands: a global review of carbon sequestration potential. Front. For. Glob. Change 8. doi: 10.3389/ffgc.2025.1648094

[B33] Varghese R. M. S. (2024). “ YOLOv8: A novel object detection algorithm with enhanced performance and robustness,” in Proceedings of the 2024 International Conference on Advances in Data Engineering and Intelligent Computing Systems ( IEEE, Chennai, India), 1–6. doi: 10.1109/ADICS58448.2024.10533619

[B34] Wang L. Cai J. Wang T. Zhao J. Gadekallu T. R. Fang K. (2024). Detection of pine wilt disease using AAV remote sensing with an improved YOLO model. IEEE J. Sel. Top. Appl. Earth Obs. Remote Sens. 17, 19230–19242. doi: 10.1109/JSTARS.2024.3478333

[B35] Wang Q. Shi Y. Suk H.-I. Suzuki K. (2017). “ Machine learning in medical imaging,” in Proceedings of the 8th International Workshop on Machine Learning in Medical Imaging ( Springer International Publishing AG, Quebec City, QC, Canada). doi: 10.1007/978-3-319-67389-9

[B36] Wu H. Xiao B. Codella N. Liu M. Dai X. Yuan L. . (2021). “ CvT: Introducing convolutions to vision transformers,” in IEEE/CVF International Conference on Computer Vision ( IEEE, Montreal, QC, Canada), 22–31. doi: 10.1109/ICCV48922.2021.00606

[B37] Xie E. Wang W. Yu Z. Anandkumar A. Alvarez J. M. Luo P. (2021). “ SegFormer: simple and efficient design for semantic segmentation with transformers,” in Proceedings of the Neural Information Processing Systems. 12077–12090, Virtual: Neural Information Processing Systems Foundation. doi: 10.5555/3540261.3541185

[B38] Xu Z. Zhou Y. Wen S. Jing W. (2025). FIDC-YOLO: improved YOLO for detecting pine wilt disease in UAV remote sensing images *via* feature interaction and dependency capturing. IEEE J. Sel. Top. Appl. Earth Obs. Remote Sens. 18, 14674–14687. doi: 10.1109/JSTARS.2025.3573741

[B39] Yao J. Song B. Chen X. Zhang M. Dong X. Liu H. . (2024). Pine-YOLO: A method for detecting pine wilt disease in unmanned aerial vehicle remote sensing images. Forests 15, 737. doi: 10.3390/f15050737

[B40] Yu R. Luo Y. Zhou Q. Zhang X. Wu D. Ren L. (2021). A machine learning algorithm to detect pine wilt disease using UAV-based hyperspectral imagery and LiDAR data at the tree level. Int. J. Appl. Earth Obs.Geoinf. 101, 102363. doi: 10.1016/j.jag.2021.102363

[B41] Yuan J. Wang L. Wang T. Bashir A. K. Al Dabel M. M. Wang J. (2025). YOLOv8-RD: high-robust pine wilt disease detection method based on residual fuzzy YOLOv8. IEEE J. Sel. Top. Appl. Earth Obs. Remote Sens. 18, 385–397. doi: 10.1109/JSTARS.2024.3494838

[B42] Zhang M. Yao J. Wang Y. Zhang Y. Lv X. Feng C. . (2025). MS-PWFOD: A model for cross-domain pine wilt disease detection. Smart Agric. Technol. 12, 101574. doi: 10.1016/j.atech.2025.101574

[B43] Zhao Z. Liao J. Zhong G. (2005). A Rapid Method for PCR Detection of Bursaphelenchus xylophilus and B. mucronatus. J. South China Agric. Univ. 26, 59–61. doi: 10.7671/j.issn.1001-411X.2005.02.015

[B44] Zhao H. Shi J. Qi X. Wang X. Jia J. (2017). “ Pyramid scene parsing network,” in IEEE Conference on Computer Vision and Pattern Recognition ( IEEE, Honolulu, HI, USA), 6230–6239. doi: 10.1109/CVPR.2017.660

[B45] Zhou B. Khosla A. Lapedriza A. Oliva A. Torralba A. (2016). “ Learning deep features for discriminative localization,” in IEEE Conference on Computer Vision and Pattern Recognition ( IEEE, Las Vegas, NV, USA), 2921–2929. doi: 10.1109/CVPR.2016.319

[B46] Zhou Z. Rahman Siddiquee M. M. Tajbakhsh N. Liang J. (2018). “ UNet++: A nested U-net architecture for medical image segmentation,” in Deep Learning in Medical Image Analysis and Multimodal Learning for Clinical Decision Support. Eds. Stoyanov D. Taylor Z. Carneiro G. Syeda-Mahmood T. Martel A. Maier-Hein L. ( Springer International Publishing, Cham), 3–11. doi: 10.1007/978-3-030-00889-5_1, PMID: PMC7329239 32613207

[B47] Zhou N. Xu M. Shen B. Hou K. Liu S. Sheng H. (2024). ViT-UNet: A vision transformer based UNet model for coastal wetland classification based on high spatial resolution imagery. IEEE J. Sel. Top. Appl. Earth Obs. Remote Sens. 17, 19575–19587. doi: 10.1109/JSTARS.2024.3487250

