# Supplementary figures and images for: MSCF-LUNet: a lightweight three-stage pine wilt disease segmentation model with multi-scale context fusion mechanism
Source: Front Plant Sci. 2026 Jan 13;16:1727626. doi: 10.3389/fpls.2025.1727626 (PMC12835376; doi:10.3389/fpls.2025.1727626)

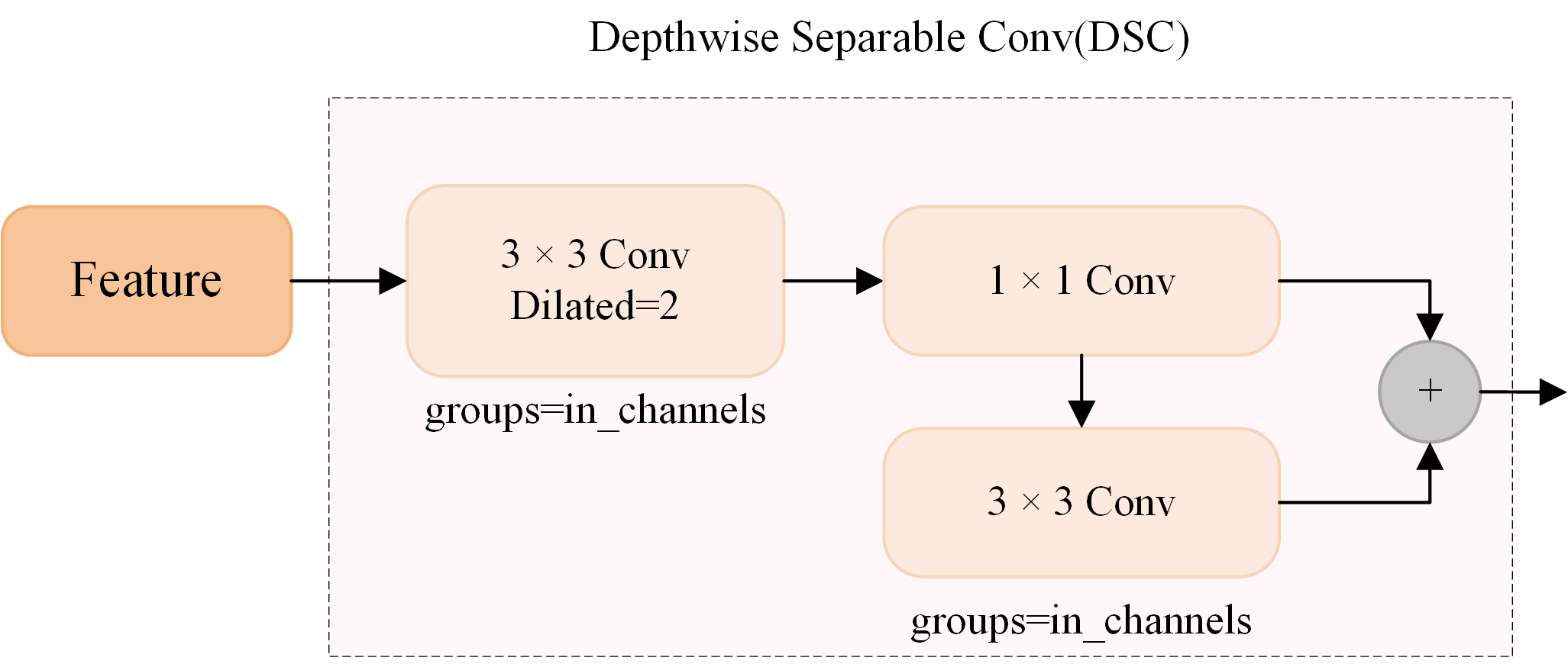

Supplement: Supplementary file 1 [file Image1.tif]

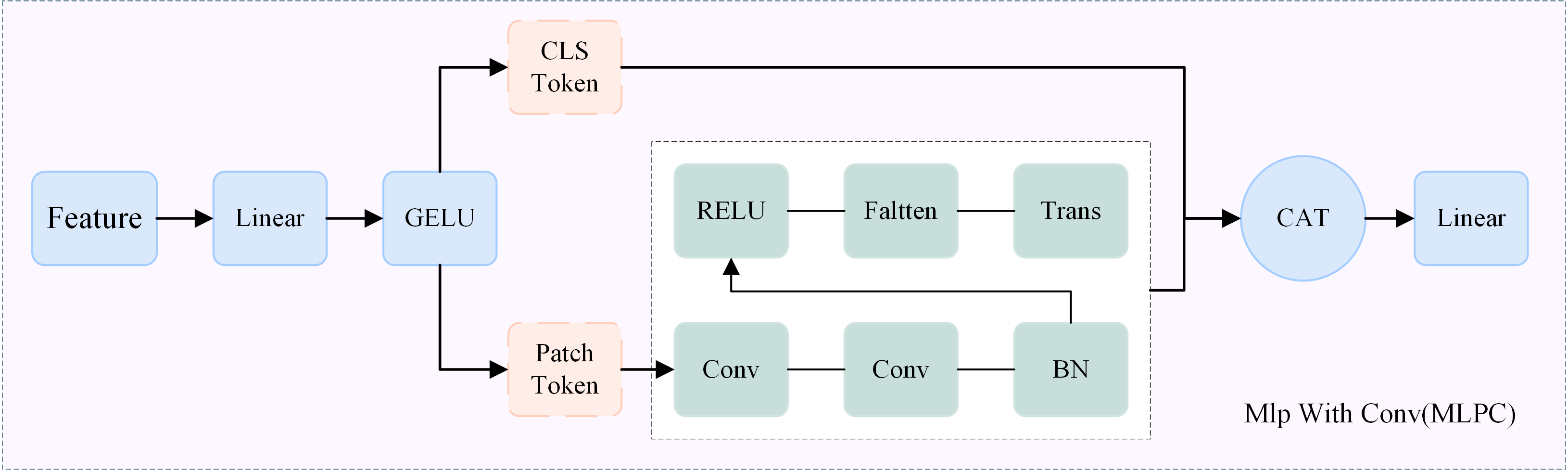

Supplement: Supplementary file 2 [file Image2.tif]

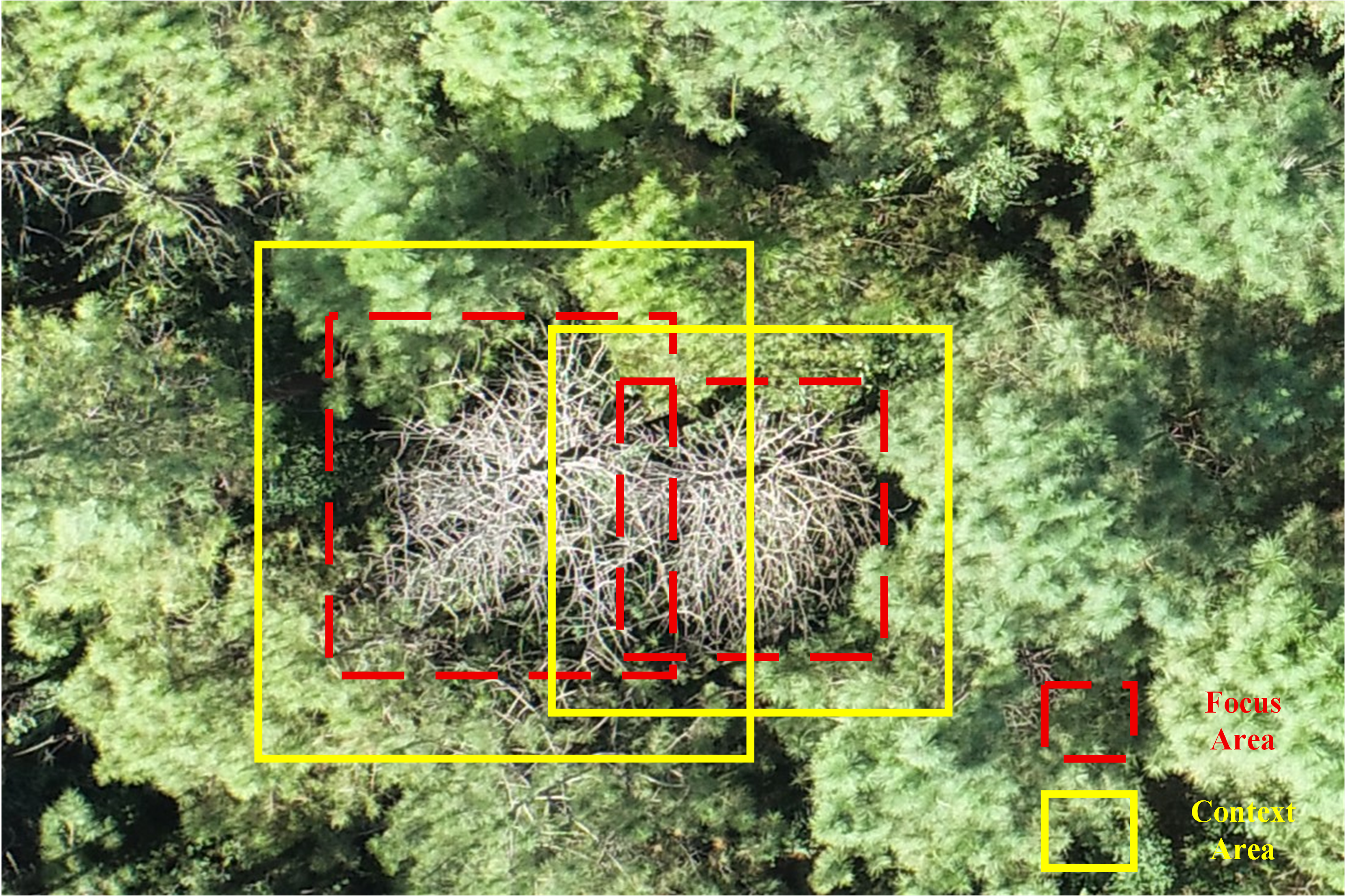

Supplement: Supplementary file 3 [file Image3.tif]
